# Exploring Blueberry Aroma Complexity by Chromatographic and Direct-Injection Spectrometric Techniques

**DOI:** 10.3389/fpls.2017.00617

**Published:** 2017-04-26

**Authors:** Brian Farneti, Iuliia Khomenko, Marcella Grisenti, Matteo Ajelli, Emanuela Betta, Alberto Alarcon Algarra, Luca Cappellin, Eugenio Aprea, Flavia Gasperi, Franco Biasioli, Lara Giongo

**Affiliations:** ^1^Genomics and Biology of Fruit Crop Department, Fondazione Edmund MachTrento, Italy; ^2^Food Quality and Nutrition Department, Fondazione Edmund MachTrento, Italy; ^3^Institut für Ionenphysik und Angewandte Physik, Leopold-Franzens Universitat InnsbruckInnsbruck, Austria

**Keywords:** *Vaccinium* spp., PTR-ToF-MS, SPME-GC-MS, VOCs, flavor, ripening, breeding

## Abstract

Blueberry (*Vaccinium* spp.) fruit consumption has increased over the last 5 years, becoming the second most important soft fruit species after strawberry. Despite the possible economic and sensory impact, the blueberry volatile organic compound (VOC) composition has been poorly investigated. Thus, the great impact of the aroma on fruit marketability stimulates the need to step forward in the understanding of this quality trait. Beside the strong effect of ripening, blueberry aroma profile also varies due to the broad genetic differences among *Vaccinium* species that have been differently introgressed in modern commercial cultivars through breeding activity. In the present study, divided into two different activities, the complexity of blueberry aroma was explored by an exhaustive untargeted VOC analysis, performed by two complementary methods: SPME-GC-MS (solid phase microextraction- gas chromatography-mass spectrometry) and PTR-ToF-MS (proton transfer reaction-time of flight-mass spectrometry). The first experiment was aimed at determining the VOC modifications during blueberry ripening for five commercially representative cultivars (“Biloxi,” “Brigitta Blue,” “Centurion,” “Chandler,” and “Ozark Blue”) harvested at four ripening stages (green, pink, ripe, and over-ripe) to outline VOCs dynamic during fruit development. The objective of the second experiment was to confirm the analytical capability of PTR-ToF-MS to profile blueberry genotypes and to identify the most characterizing VOCs. In this case, 11 accessions belonging to different *Vaccinium* species were employed: *V*. *corymbosum* L. (“Brigitta,” “Chandler,” “Liberty,” and “Ozark Blue”), *V. virgatum* Aiton (“Centurion,” “Powder Blue,” and “Sky Blue”), *V. myrtillus* L. (three wild genotypes of different mountain locations), and one accession of *V. cylindraceum* Smith. This comprehensive characterization of blueberry aroma allowed the identification of a wide pull of VOCs, for the most aldehydes, alcohols, terpenoids, and esters that can be used as putative biomarkers to rapidly evaluate the blueberry aroma variations related to ripening and/or senescence as well as to genetic background differences. Moreover, the obtained results demonstrated the complementarity between chromatographic and direct-injection mass spectrometric techniques to study the blueberry aroma.

## Introduction

The quality of fruits has to be considered as a central trait to address consumer appreciation and optimize the whole production chain management (Costa et al., 2000; Mowat and Collins, 2000; Benner and Geerts, 2003; Klee, 2010). In order to satisfy consumer's demands more effort has to be devoted to improve and optimize quality upon delivery to consumers. Quality of a fresh product can be defined by the achievement of four principal quality elements: appearance, flavor, texture, and nutritional properties (Costa et al., 2011). Defining and quantifying these quality components, in relation with distinct segments of the production chain, needs comprehensive investigations and a tight synergy of analytical approaches with a particular focus on rapid and non-invasive methods.

For many years most breeding efforts have been primarily devoted to improve and maintain the external quality of fruits, with little attention to other intrinsic characteristics. Selection for yield, fruit size, color, and shelf life traits might have had unintended negative consequences on sensory quality and nutritional effects (Goff and Klee, 2006; Farneti et al., 2015a, 2017; Tieman et al., 2017). Fruit flavor, in particular, depends upon taste (balance between sweetness and sourness or acidity, and low or no astringency) and aroma (concentration of VOCs). Although taste and aroma are well-integrated in their contribution to the overall flavor, aroma is often considered playing a dominant role (Folta and Klee, 2016). Although a single fruit synthesizes several 100 volatiles, only a small subset generates the “flavor fingerprint” that helps animals and humans to recognize appropriate and avoid dangerous food (Goff and Klee, 2006). Since aroma involves the perception of a myriad of VOCs, their assessment would be crucial to guarantee the selection and marketability of high-quality fruits. Another aspect to take into account is the interaction of volatile compounds may have with taste in fruits as recently evidenced in apple sweetness perception (Aprea et al., 2017). In the near future, the main breeding goal will be to produce fruits and vegetables that consumers actively seek, while maintaining industry-mandated qualities. High priority should thus be given to replacing poor flavor cultivars with more favorable ones, exploiting the variability already available in nature (Folta and Klee, 2016).

The extraordinary nutraceutical properties (Norberto et al., 2013) and the unique flavor (Gilbert et al., 2014, 2015), are the chief quality traits that are swiftly enhancing blueberry (*Vaccinium* spp.) consumption. Worldwide blueberry production has indeed increased over the last decade (Brazelton, 2011; Payne, 2014; Clarke, 2016), becoming the second most important soft fruit species after strawberry. Despite the economic and nutraceutical importance of blueberry, there has been little mention in the literature, over last 10 years, of the VOC composition of this fruit and of its possible impact on consumer preferences.

Blueberry aroma depends on the interaction of dozens of VOCs (Du et al., 2011; Gilbert et al., 2013; Beaulieu et al., 2014; Gilbert et al., 2015) synthesized by the fruit during ripening. Among them only a minor set of chemical compounds, for the most aldehydes, alcohols, ketones, terpenoids, and esters can be distinctly perceived at the sensorial level (Du and Rouseff, 2014; Gilbert et al., 2015). Despite the strong effect of ripening, the blueberry aroma profiling also varies due to the broad genetic differences among the *Vaccinium* species. For instance lowbush blueberry (*V. angustifolium* L.), bilberry (*V. myrtillus* L.), and other wild species are mostly characterized by a high production of esters (i.e., methyl acetate, ethyl acetate, or methyl butanoate) while highbush (*V. corymbosum* L.) and rabbiteye blueberry (*V. virgatum* Aiton) profiles are mostly characterized by a high concentration of “green compounds” such as (E)-2-hexenal, hexanal, and (Z)-3-hexenol and terpene alcohols such as linalool, nerol, and geraniol (von Sydow and Anjou, 1969; von Sydow et al., 1970; Hall et al., 1979; Horvat et al., 1983, 1996).

Aroma characterization of different species has always represented a main analytical issue, especially because wide sample sets are needed to cover the expected biological variability. Traditionally, flavor attributes of horticultural products are assessed by sensory panels. However, this procedure is time consuming, and expensive. Therefore, in practical contexts, high resolution and rapid screening techniques are needed as analytical support for sensory analysis. These analytical tools have to deal with important issues such as the need of separating and quantifying VOCs in complex gas mixtures and the simultaneous detection of concentrations which may span a large range, from trace levels (i.e., part per trillion) to parts per million (Biasioli et al., 2011).

Given these experimental constraints, the ideal methodology for VOCs monitoring should be highly selective, with high sensitivity and dynamic range, and with high time resolution. The benchmark analytical method for VOCs identification and quantification is currently gas chromatography-mass spectrometry (GC-MS), often coupled with solid-phase microextraction (e.g., SPME fibers) to lower the detection limits. Although valuable and, in many cases, indispensable, gas chromatographic headspace analyses have several disadvantages such as low time resolution, laborious sample preparation and long operation time particularly when a concentrate headspace VOC content is needed to improve the detection limit (Dewulf et al., 2002). Moreover, the application of thermal desorption units, such as SPME fibers, precludes a feasible quantitative analysis of multicomponent mixtures, since the competition for active sites on the fiber and the relative proportions of the adsorbed compounds depend on their ratio in the sample VOC headspace (Górecki et al., 1999). Overcoming such limits means employing techniques without chromatographic separation. This might be the reason why total aromatic volatile concentrations, collected and concentrated from blueberry fruit extracts using a SPME technique, were not strongly correlated with sensory scores for flavor, overall eating quality or to any other sensory characteristic (Saftner et al., 2008; Gilbert et al., 2015). Thus, volatile concentration, at least when analyzed on the headspace of intact fruit by SPME technique, might not be a good indicator of blueberry taste or overall eating quality.

Different methods have been recently proposed, such as arrays of solid-state gas sensors (E-Noses), and direct injection mass spectrometry (DI-MS; Biasioli et al., 2011). Besides its technological performances (e.g., sensitivity and selectivity), the greatest difficulty arising in DI-MS technologies, due to the lack of chromatographic separation, is the need to identify compounds that generate the observed peaks, since the latter can be the results of overlapping signals from the mix of different VOCs present in the sample. Among DI-MS techniques, Proton Transfer Reaction-Mass Spectrometry (PTR-MS) has the advantage of a very low detection limit and high sensitivity. (Blake et al., 2009). Significant improvements have been made by coupling PTR-MS technology with Time-of-Flight mass spectrometry (ToF-MS). PTR-ToF-MS instruments can generate entire mass spectra of complex trace gas mixtures in short response times with high mass resolution and with virtually no upper mass limit (Jordan et al., 2009).

In the present study the complexity of blueberry aroma was explored by an exhaustive untargeted VOC analysis, done by SPME-GC-MS and PTR-ToF-MS analysis. The aim of this investigation was to acquire a detailed and comprehensive characterization of the blueberry aroma according to different ripening stages and genetic differences, as well as to investigate the potential of PTR-ToF-MS as a rapid and reliable technique to address issues related to blueberry quality.

## Materials and methods

### Plant materials

In this investigation, blueberry accessions were chosen from the experimental field of FEM Research and Innovation Center at Pergine (Trento), located in the north of Italy (Trentino Alto Adige region). At the time of the analysis, plants were in the full production phase, between 7 and 10 years old. The bushes were grown in trenches lined with permeable tissue, adjusted with a pit-bark mix. The crop's frame was 2.5 m between rows and 1 m along the row. An automatic fertigation system was used to guarantee water supply and soil acidification at pH 4.5 (by adding nitric acid 52% with an automatic dispenser), while water conductivity was periodically monitored at 1300 μS with a conductivimeter (Crison Instrument Mod. CM35). Bushes were maintained following standard pruning and surface bark mulching renewal. In the plot, each of the accessions was represented by five plants.

For the first experiment, aimed at determining the VOCs modifications during fruit ripening, we employed five blueberry cultivars, namely “Biloxi,” “Brigitta Blue,” “Centurion,” “Chandler,” and “Ozark Blue.”

For the second experiment, aimed at testing the analytical capacity of PTR-ToF-MS to profile blueberry genotypes based on VOCs, we employed eleven accessions of four different *Vaccinium* species: *V*. *corymbosum* L. (“Brigitta Blue,” “Chandler,” “Liberty,” and “Ozark Blue”), *V. virgatum* Aiton (“Centurion,” “Powder Blue,” and “Sky Blue”), *V. myrtillus* L. (three genotypes of different mountain locations), and one accession of *V. cylindraceum* Smith.

### Fruit maturity assessment

Blueberry fruits, free from external damages or irregularities, were harvested from multiple plants an sorted into the established ripening stages [green (G), pink (P), ripe (R), and overripe (Or)] analytically determined using Minolta colorimeter and non-destructive compression test using FirmTech firmness tester (BioWorks, Wamengo, KS, USA). Ten homogeneous berries, for each ripening stage, were selected for fruit maturity assessment. Texture was profiled by a texture analyzer (Zwick Roell, Italy), which profiled a mechanical force displacement using a 5 kg loading cell and a cylindrical flat head probe with a diameter of 4 mm entering into the berry flesh from the sagittal side (for more details see Giongo et al., 2013). On the force displacement profile, seven parameters were considered: maximum force, final force, area, maximum deformation, minimum deformation, maximum force strain, and gradient (or Young's module, also known as the elasticity module). Total soluble solid (TSS, °Bx), pH, and titratable acidity (TA, meq/100 g FW) were assessed on homogeneous berries with a DBR35 refractometer (XS Instruments, 199 Poncarale, Brescia, Italy), pH Meter, and Compact Titrator (Crison Instruments S.A., Alella, Barcelona, 200 Spain), respectively.

### VOC analysis by SPME-GC-MS

Three replicates of 1.0 g of powdered frozen samples, conserved at −80°C, were immediately inserted into 20 ml glass vials equipped with PTFE/silicone septa (Agilent, Cernusco sul Naviglio, Italy) and mixed with 1.0 ml of deionized water, 400 mg of sodium chloride, 5 mg of ascorbic acid, and 5 mg of citric acid (for more details see Aprea et al., 2011). Samples were then preserved at 4°C till the analysis.

The vials were equilibrated at 40°C for 10 min with constant stirring. Solid-phase microextraction fiber (DVB/CAR/PDMS, Supelco, Bellefonte, PA, USA) was exposed for 30 min in the vial headspace. The compounds adsorbed by HS-SPME were analyzed with a GC interfaced with a mass detector operating in electron ionization (EI) mode (internal ionization source; 70 eV) with a scan range of *m/z* 33–350 (GC Clarus 500, PerkinElmer, Norwalk CT, USA). Separation was carried out in an HP-INNOWax fused silica capillary column (30 m, 0.32-mm ID, 0.5-μm film thickness; Agilent Technologies, Palo Alto, CA, USA). The initial GC oven temperature was 40°C rising to 220°C at 4°C min^−1^, the temperature of 220°C was maintained for 1 min, then increased at 10°C min^−1^ until it reached 250°C, which was maintained for 1 min. The carrier gas was helium at a constant column flow rate of 1.5 ml min^−1^. Samples were analyzed in triplicates. Semiquantitative data were expressed as microgram per liter equivalent of 2-octanol. Compound identification was based on mass spectra matching with the standard NIST/EPA/NIH (NIST 14) and Wiley 7th Mass Spectral Libraries, and linear retention indices (LRI) compared with the literature. LRI were calculated under the same chromatographic conditions after injection of a C7–C30 n-alkane series (Supelco).

### VOC analysis by PTR-ToF-MS

Measurements of blueberry VOCs with a PTR-ToF-MS 8000 apparatus (Ionicon Analytik GmbH, Innsbruck, Austria) were performed in three sample replicates prepared as for SPME-GC-MS analysis (without adding the internal standard). The conditions in the drift tube were the following ones: 110°C drift tube temperature, 2.30 mbar drift pressure, 550 V drift voltage. This leads to an E/N ratio of about 140 Townsend (Td), with E corresponding to the electric field strength and N to the gas number density (1 Td = 1017 Vcm^2^). The sampling time per channel of ToF acquisition was 0.1 ns, amounting to 350,000 channels for a mass spectrum ranging up to *m/z* = 400. Every single spectrum is the sum of about 28.600 acquisitions, resulting in a time resolution of 1 s. Sampling was performed in 60 cycles resulting in an analysis time of 60 s/sample. Each measurement was conducted automatically after 20 min of sample incubation at 40°C by using an adapted GC autosampler (MPS Multipurpose Sampler, GERSTEL) and it lasted for about 2 min (Capozzi et al., 2017). During each measurement a sample headspace was withdrawn through PTR-MS inlet with 40 sccm flow. For prevention of low pressure inside the vial, zero air was flushed continuously through it.

The analysis of PTR-ToF-MS spectral data proceeded as follows. Count losses due to the ion detector dead time were corrected off-line via a methodology based on Poisson statistics (Titzmann et al., 2010). To reach a good mass accuracy (up to 0.001 Th), internal calibration was performed according to a procedure described by Cappellin et al. (2011). Noise reduction, baseline removal and peak intensity extraction were performed according to Cappellin et al. (2011), using modified Gaussians to fit the peaks. Absolute headspace VOC concentrations expressed in ppbv (parts per billion by volume) were calculated from peak intensities according to Cappellin et al. (2012b).

### Statistical analysis

The detection of the array of masses with PTR-ToF-MS was reduced by applying noise and correlation coefficient thresholds. The first removed peaks not significantly different from blank samples (Farneti et al., 2015b); the latter excluded peaks having over 99% correlation, which correspond for the most to isotopes of monoisotopic masses (Farneti et al., 2017).

R.3.2.2 internal statistical functions and the external packages “mixOmics” and “clValid” were used for the multivariate statistical methods [Principal Component Analysis (PCA), Partial Least Squares (PLS), Self Organizing Tree Algorithm (SOTA)] employed in the whole work.

Visualization of significant VOCs correlations (*P* < 0.01; *R* > 0.60) was conducted by the generation of a PLS regression Network with Cytoscape (version 3.2.1; Cline et al., 2007).

## Results

### Fruit ripening assessment

Fruits were non-destructively sorted into four ripening classes based on color. The homogeneity of each fruit batch was successively confirmed by destructive quality assessments such as texture, pH, titratable acidity, and soluble solid content, on randomly picked fruits.

Principal component analysis based on fruit textural proprieties (Supplementary Figure 1), beyond a distinct separation of *Vaccinium* cultivars, revealed a clear separation of the four harvest ripening stages, mostly explained by the variability of the first principal component (PC1: 95.6%). More ripe fruits were characterized by a greater deformation due to the applied forces while more unripe fruit had a higher resistance to the forces (F_Min and F_Max) that resulted also in a greater area under the deformation curve.

As expected, textural differences between ripe and over ripe fruits were not as discernible as for the more unripe (green and pink) ones. However, differences between these two classes were magnified by the results of pH, treatable acidity (TA), and total soluble solids (TSS; Supplementary Figure 2). Overripe fruits of each cultivar were indeed characterized by higher pH and TSS-values and lower TA.

### Chemical composition of blueberry aroma assessed by SPME-GC-MS

The gas chromatographic analysis by SPME-GC-MS assessed on five *Vaccinium* cultivars (“Biloxi,” “Brigitta Blue,” “Centurion,” “Chandler,” and “Ozark Blue”) harvested at different ripening stages (green, pink, ripe, over ripe) allowed the detection of 106 VOCs, among which only six were not identified (reported as “Unknown”; Table 1). Esters, 25 in total, were the most represented chemical class. Other classes of compounds are aldehydes (18 compounds), alcohols (16), monoterpenes (14), ketones (7), acids (4), hydrocarbons (4), sesquiterpenes (3), lactones (1), and norisoprenoids (1).

**Table 1:** **Volatile compounds detected by SPME-GC-MS in blueberry fruits at different ripening stages**.

**Name**	**ID**	**SOTA^a^**	**Formula**	**RT**	**KI Calc**	**KI Nist**	**Min^b^**	**Max^b^**	**Mean^b^**
**ACIDS**									
Hexanoic acid	Ac_1	8	C_6_H_12_O_2_	33.27	2,044	1,846	0.1	6.9	0.6
Octanoic acid	Ac_2	8	C_8_H_16_O_2_	38.25	2,222	2,060	0.0	3.0	0.4
Nonanoic acid	Ac_3	3	C_9_H_18_O_2_	40.40	2,299	2,171	1.5	8.4	3.4
Decanoic acid	Ac_4	8	C_10_H_20_O_2_	42.77	2,384	2,276	1.2	4.9	2.9
**ALCOHOLS**									
Ethanol	Al_1	1	C_2_H_6_O	2.36	937	932	569.6	809.2	675.2
3-Methyl-1-butanol	Al_2	1	C_5_H_12_O	9.21	1,223	1,209	0.0	2.8	0.5
Pentanol	Al_3	2	C_5_H_12_O	10.57	1,264	1,250	1.9	12.1	6.0
2-Heptanol	Al_4	1	C_7_H_16_O	12.88	1,332	1,320	0.0	1.6	0.1
Hexanol	Al_5	8	C_6_H_14_O	13.92	1,363	1,355	3.0	272.3	26.4
(E)-3-hexen-1-ol	Al_6	4	C_6_H_12_O	14.26	1,373	1,367	0.0	2.5	0.6
(Z)-3-hexen-1-ol	Al_7	1	C_6_H_12_O	14.90	1,392	1,382	1.1	147.1	34.4
(Z)-2-hexen-1-ol	Al_8	6	C_6_H_12_O	15.63	1,415	1,416	2.8	119.8	30.5
1-Octen-3-ol	Al_9	6	C_8_H_16_O	17.05	1,460	1,450	1.8	5.3	3.4
1-Heptanol	Al_10	8	C_7_H_16_O	17.17	1,464	1,453	0.5	11.2	1.2
2-Ethyl-1-hexanol	Al_11	1	C_8_H_18_O	18.26	1,498	1,491	0.4	1.1	0.6
1-Octanol	Al_12	8	C_8_H_18_O	20.30	1,566	1,557	1.1	28.2	3.0
HO-trienol	Al_13	1	C_10_H_16_O	21.86	1,619	1,613	0.0	8.1	1.1
1-Nonanol	Al_14	8	C_9_H_20_O	23.26	1,667	1,660	0.5	4.7	1.1
Benzyl alcohol	Al_15	8	C_7_H_8_O	29.01	1,879	1,870	0.0	3.9	0.4
Phenetyl alcohol	Al_16	8	C_8_H_10_O	29.85	1,912	1,906	0.0	1.5	0.1
**ALDEHYDES**									
2-Methyl butanal+3-methyl butanal	Ad_1	1	C_5_H_10_O	3.03	984	914	1.8	17.3	8.5
Hexanal	Ad_2	6	C_6_H_12_O	5.33	1,097	1,083	44.7	287.2	134.4
(E)-2-pentenal	Ad_3	1	C_5_H_8_O	6.66	1,142	1,127	0.5	11.1	3.4
(Z)-3-hexenal	Ad_4	1	C_6_H_10_O	7.68	1,175	1,141	1.6	508.7	176.3
Heptanal	Ad_5	8	C_7_H_14_O	8.39	1,198	1,184	2.8	15.0	4.7
(Z)-2-hexenal	Ad_6	1	C_6_H_10_O	8.91	1,214	1,189	6.2	45.9	25.8
(E)-2-hexenal	Ad_7	5	C_6_H_10_O	9.42	1,229	1,216	201.8	1206.9	632.4
Octanal	Ad_8	3	C_8_H_16_O	11.74	1,298	1,289	2.5	7.3	4.6
(E)-2-heptenal	Ad_9	1	C_7_H_12_O	12.80	1,330	1,323	3.9	17.8	10.7
2-Nonenal	Ad_10	3	C_9_H_16_O	15.13	1,399	1,537	4.3	14.2	8.1
(E,Z)-2,4-hexadienal	Ad_11	1	C_6_H_8_O	15.30	1,404	1,391	0.5	8.1	3.6
(E,E)-2,4-hexadienal	Ad_12	1	C_6_H_8_O	15.39	1,407	1,395	3.3	40.3	17.8
(E)-2-octenal	Ad_13	3	C_8_H_14_O	16.17	1,432	1,429	6.5	13.6	9.7
(E,E)-2,4-heptadienal	Ad_14	1	C_7_H_10_O	17.37	1,470	1,495	0.2	2.2	0.9
Decanal	Ad_15	8	C_10_H_20_O	18.40	1,503	1,498	1.0	4.2	2.3
Benzaldehyde	Ad_16	1	C_7_H_6_O	19.04	1,524	1,520	0.6	2.8	1.2
(E)-2-nonenal	Ad_17	7	C_9_H_16_O	19.44	1,538	1,534	0.6	3.4	1.5
3-Ethyl benzaldehyde	Ad_18	8	C_9_H_10_O	24.37	1,706	1,698	0.0	0.9	0.2
**ESTERS**									
Methyl acetate	E_1	8	C_3_H_6_O_2_	1.40	829	828	0.0	2.9	0.2
Ethyl acetate	E_2	8	C_4_H_8_O_2_	1.83	897	888	0.9	45.4	5.4
Ethyl propanoate	E_3	8	C_5_H_10_O_2_	2.68	960	953	0.0	0.3	0.0
Ethyl isobutanoate	E_4	8	C_6_H_12_O_2_	2.80	968	961	0.0	0.3	0.0
Methyl-2-methyl butanoate	E_5	8	C_6_H_12_O_2_	3.64	1,018	1,009	0.0	2.3	0.1
Methyl isovalerate	E_6	8	C_6_H_12_O_2_	3.85	1,028	1,018	0.0	20.0	1.9
Ethyl butyrate	E_7	8	C_6_H_12_O_2_	4.28	1,048	1,035	0.0	0.3	0.0
Ethyl-2-methyl butanoate	E_8	8	C_7_H_14_O_2_	4.60	1,063	1,051	0.0	8.9	0.7
Ethyl isovalerate	E_9	8	C_7_H_14_O_2_	4.99	1,081	1,068	0.0	76.1	8.0
Ethyl (2E)-2-butenoate	E_10	8	C_6_H_10_O_2_	7.78	1,178	1,160	0.0	3.3	0.3
Ethyl hexanoate	E_11	3	C_8_H_16_O_2_	10.01	1,247	1,233	0.0	0.2	0.0
Hexyl acetate	E_12	8	C_8_H_16_O_2_	11.27	1,284	1,272	0.0	3.4	0.4
(Z)-3-hexenyl acetate	E_13	1	C_8_H_14_O_2_	12.75	1,328	1,315	0.0	195.0	22.9
2-Hexenyl acetate	E_14	7	C_8_H_14_O_2_	13.33	1,345	1,352	0.0	18.5	2.5
Methyl 3-hydroxy-3-methylbutanoate	E_15	8	C_6_H_12_O_3_	14.44	1,379	1,363	0.0	3.7	0.2
Ethyl-3-hydroxy-3-methylbutanoate	E_16	1	C_7_H_14_O_3_	15.65	1,416	1,404	0.0	1.1	0.1
Ethyl-2-hydroxy-3-methylbutanoate	E_17	8	C_7_H_14_O_3_	16.15	1,432	1,422	0.0	4.9	0.4
(E,Z)-ethyl 2,4-hexadienoate	E_18	1	C_10_H_18_O_2_	16.90	1,455		0.0	0.3	0.0
(Z)-3-hexenyl butanoate	E_19	1	C_10_H_18_O_2_	17.29	1,468	1,454	0.0	13.2	1.2
(E,E)-ethyl 2,4-hexadienoate	E_20	2	C_10_H_12_O_2_	18.00	1,490	1,501	0.0	6.1	0.8
(Z,Z)-ethyl 2,4-hexadienoate	E_21	2	C_8_H_12_O_2_	18.70	1,513		0.0	1.2	0.2
Ethyl furan-2-carboxylate	E_22	1	C_7_H_8_O_3_	22.16	1,629	1,618	0.0	0.3	0.0
Ethyl benzoate	E_23	2	C_9_H_10_O_2_	23.26	1,667	1,658	0.0	0.5	0.1
Ethyl phenyl acetate	E_24	1	C_9_H_10_O_2_	26.63	1,789	1,783	0.0	0.1	0.0
2-Ethyl hexyl salicylate	E_25	8	C_15_H_22_O_3_	38.86	2,244		0.0	16.5	2.0
**HYDROCARBONS**									
Octane	H_1	7	C_8_H_18_	1.23	802	800	0.2	23.5	5.2
Ethyl benzene	H_2	8	C_8_H_10_	6.48	1,136	1,125	0.6	8.2	3.5
p-Xylene	H_3	8	C_8_H_10_	6.71	1,143	1,127	0.0	1.3	0.5
m-Xylene	H_4	8	C_8_H_10_	6.90	1,150	1,132	0.0	2.9	1.2
***Ketones***									
2-Heptanone	K_1	1	C_7_H_14_O	8.27	1,195	1,182	0.0	140.9	16.7
2-Octanone	K_2	2	C_8_H_16_O	11.59	1,294	1,287	3.4	4.5	3.8
1-Octen-3-one	K_3	3	C_8_H_14_O	12.16	1,311	1,300	0.9	4.1	2.3
6-Methyl-5-hepten-2-one	K_4	4	C_8_H_14_O	13.34	1,346	1,338	14.4	52.1	30.0
2-Nonanone	K_5	1	C_9_H_18_O	14.96	1,394	1,390	0.0	11.6	2.7
2-Undecanone	K_6	1	C_11_H_22_O	21.38	1,602	1,598	0.0	7.6	2.3
Acetophenone	K_7	3	C_8_H_8_O	22.76	1,650	1,647	0.1	2.4	0.5
**LACTONES**									
Butyrolactone	L_1	7	C_4_H_6_O_2_	22.05	1,625	1,632	0.6	2.0	1.0
**MONOTERPENES**									
β-Myrcene	M_1	7	C_10_H_16_	7.68	1,175	1,161	0.0	8.7	1.6
Limonene	M_2	7	C_10_H_16_	8.60	1,205	1,200	0.3	35.2	8.7
1,8-Cineole	M_3	1	C_10_H_18_O	8.83	1,212	1,213	4.0	222.4	63.6
(E)-β-ocimene	M_4	7	C_10_H_16_	10.54	1,262	1,250	0.0	2.5	1.0
α-Terpinolene	M_5	7	C_10_H_16_	11.41	1,288	1,283	0.5	8.0	2.9
Linalool oxide A	M_6	1	C_10_H_18_O_2_	16.55	1,444	1,452	0.1	5.9	1.6
Linalool oxide B	M_7	1	C_10_H_18_O_2_	17.46	1,473	1,444	0.0	14.3	2.9
Linalool	M_8	6	C_10_H_18_O	20.03	1,557	1,547	11.6	193.2	105.7
4-Terpineol	M_9	1	C_10_H_18_O	21.41	1,603	1,602	0.0	14.1	1.0
α-Terpineol	M_10	7	C_10_H_18_O	24.18	1,699	1,697	2.0	17.0	9.8
Nerol	M_11	8	C_10_H_18_O	27.08	1,806	1,797	0.0	1.5	0.3
Geraniol	M_12	7	C_10_H_18_O	28.36	1,855	1,847	0.3	7.2	1.9
Geranyl acetone	M_13	6	C_13_H_22_O	28.34	1,854	1,859	1.6	28.1	9.3
Eugenol	M_14	8	C_10_H_12_O_2_	36.07	2,144	2,169	0.0	2.4	0.1
**NORISOPRENOIDS**									
β-Damascenone	N_1	2	C_13_H_18_O	27.43	1,819	1,823	0.0	0.3	0.1
**SESQUITERPENES**									
δ-Elemene	S_1	8	C_15_H_24_	17.32	1,469	1,470	0.0	2.4	0.1
(E)-caryophyllene	S_2	1	C_15_H_24_	21.02	1,590	1,595	0.0	8.3	1.2
Caryophyllene oxide	S_3	1	C_15_H_24_O	31.31	1,970	1,989	0.0	1.5	0.3
**UNIDENTIFIED COMPOUNDS**									
Unknown 1	U_1	2	#	7.98	1,185		0.0	6.5	0.6
Unknown 2	U_2	7	#	10.18	1,252		0.0	84.2	18.4
Unknown 3	U_3	8	#	12.27	1,314		0.0	0.4	0.0
Unknown 4	U_4	7	#	12.57	1,323		0.0	40.6	4.7
Unknown 5	U_5	6	#	19.18	1,529		0.3	2.3	1.1
Unknown 6	U_6	1	#	31.36	1,972		0.2	8.6	2.7

a *SOTA (self-organizing tree algorithm) clusters based on Figure 2*.

b *μg/Kg of 2-octanol*.

# *MS detection spectra showed in Supplementary Figure 3*.

**Figure 1:**
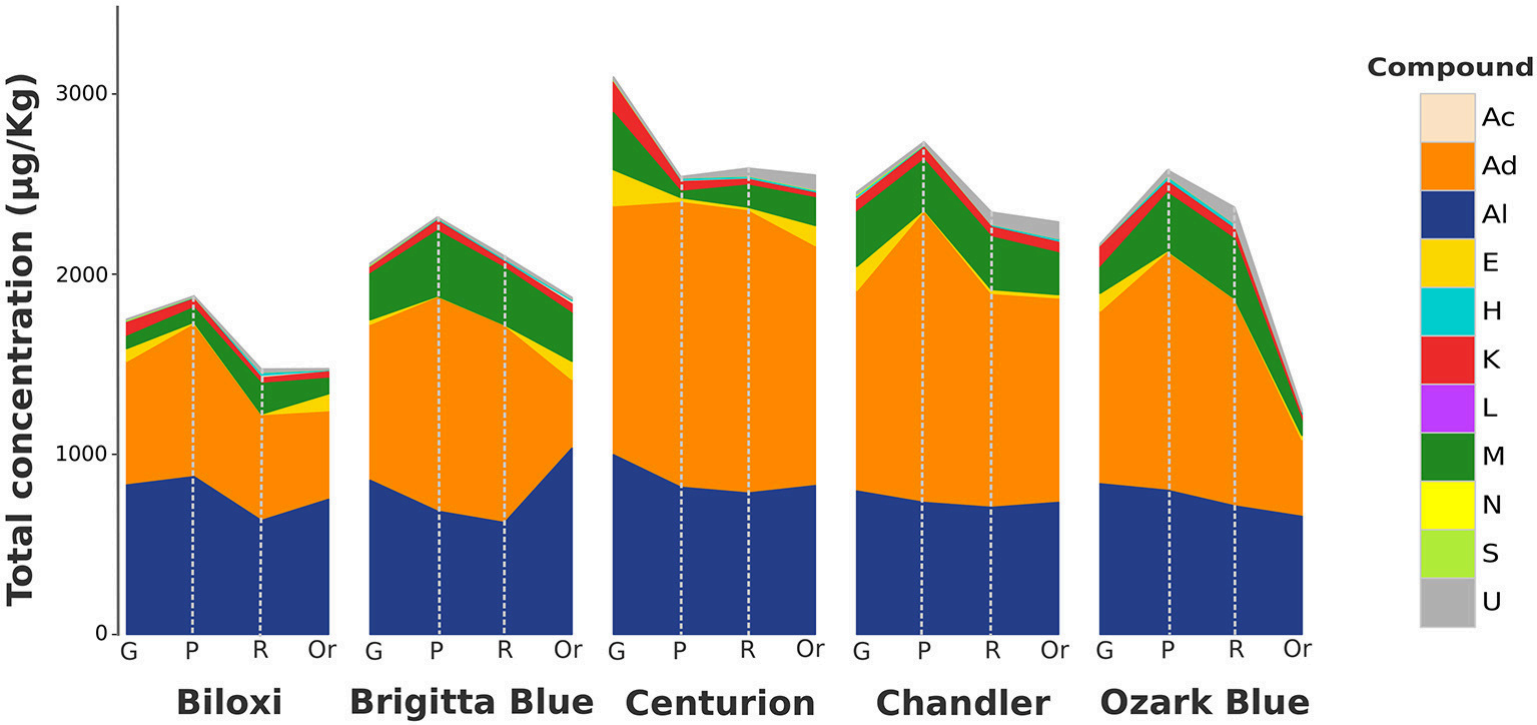
**Stacked area chart of the total VOC content of blueberry during fruit ripening assessed by SPME-GC-MS analysis**. The total VOC concentration, expressed as μg/Kg of 2-octanol, is reported for each blueberry cultivar (“Biloxi,” “Brigitta Blue,” “Centurion,” “Chandler,” and “Ozark Blue”) at four ripening stages [green (G), pink (P), ripe (R), and overripe (Or)]. Each VOC classes [acids (Ac), aldehydes (Ad), alcohols (Al), esters (E), hydrocarbons (H), ketones (K), lactones (L), monoterpenes (M), norisoprenoids (N), sesquiterpenes (S), unknowns (U)] is described with a different color.

Based on VOC relative concentration, aldehydes were the most abundant class (in terms of total chromatographic area) since they covered almost 50% of the overall *Vaccinium* volatile profile (Figure 1). The highest fraction of aldehydes was composed by C6 aldehydes such as (E)-2-hexenal, hexanal, (Z)-3-hexenal, hexadienal, or heptenal. These compounds, predominantly detected in unripe fruits, decreased during fruit ripening with slight variability between cultivars. Remarkably, some isomers of these aldehydes, such as (Z)-3-hexenal and (E)-2-hexenal, did not show the same evolution during fruit ripening. For instance, while (Z)-3-hexenal concentration decreased exponentially during ripening, (E)-2-hexenal concentration reached the utmost level at the pink stage and it lasted till the full ripe stage. Hexanal, the third aldehyde based on average concentration levels, after (E)-2-hexenal and (Z)-3-hexenal, revealed a production trend similar to (E)-2-hexenal one.

Over 35% of the blueberry chromatographic profile was determined by alcoholic compounds (Figure 1). Nevertheless, this result should be discreetly considered since the largest fraction of these compounds was covered by ethanol. Besides ethanol, (Z)-3-hexenol and (Z)-2-hexenol were the most abundant alcohols. These alcohols, synthesized from their corresponding aldehydes [(Z)-3-hexenal and (Z)-2-hexenal], revealed an opposite evolution during fruit ripening. (Z)-3-hexanol was mostly synthesized by green blueberry and it suddenly decreased during fruit ripening. (Z)-2-hexenol amount, instead, increased linearly during fruit ripening and it reached significantly different end-levels at the overripe stage, according to the genotype. Likewise, hexanol was synthesized during fruit ripening and it reached different concentration that were genotype specific; for instance fruits of “Brigitta Blue,” differently from the other four cultivars considered in this study, were characterized by an extremely high amount of hexanol in the overripe stage.

Another important fraction of the blueberry volatile profile was composed by monoterpenes (Figure 1), being 1,8-cineole and linalool two main elements of this chemical family: 1,8-cineole was mostly synthesized in green fruit and it rapidly reduced during fruit ripening; linalool was mostly produced in fruit at pink stage and preserved during the last phases of fruit ripening with different end-level amount according to the cultivar. A production course similar to the one of linalool was found also for geranyl acetone. Most of the other terpenes detected in blueberry, such as limonene, α-terpinolene, or α-terpineol, were mostly synthesized between the pink and ripe stage.

Esters, although been present at lower average amount compared to the aforementioned compounds (Figure 1), had an important role to fully characterized the blueberry aroma, mostly at the overripe maturity stage. Most of the esters, such as ethyl acetate, methyl isovalerate, or ethyl isovalerate were largely synthesized in overripe fruit, while, contrariwise, only few esters, for instance (Z)-3-hexenyl acetate, were detectable at high concentration in green fruit.

Seven ketones were detected in blueberry fruit. 2-heptanone and 6-methyl-5-hepten-2-one were the two molecules with the higher chromatographic area. 2-heptanone, as well as 2-undecanone and 2-nonanone, were mostly detected in green fruit, with significant amount differences among cultivars. Their content decreased till trace levels during fruit ripening. 6-methyl-5-hepten-2-one was instead detectable in blueberry in all ripening stages without any distinct course related to fruit ripening.

Four hydrocarbons (octane, ethyl benzene, p-xylene, and m-xylene) were identified and, even if present in all ripening stages, they were mostly expressed in the ripe and overripe stages.

The remaining fraction of the blueberry aroma profile was composed by four volatile acids (hexanoic acid, octanoic acid, nonanoic acid, and decanoic acid), three sesquiterpenes (δ-elemene, (E)-caryophyllene and caryophyllene oxide), one norisoprenoid (β-damascenone), and one lactone (butyrolactone). Apart from caryophyllene, for the most produced only in green fruits, all these compounds were stable during all ripening phases except for the overripe fruits of “Brigitta Blue” that were characterized by an increased level of hexanoic acid and octanoic acid content.

#### Effect of ripening and genetic differences on the blueberry aroma profile

**Figure 2:**
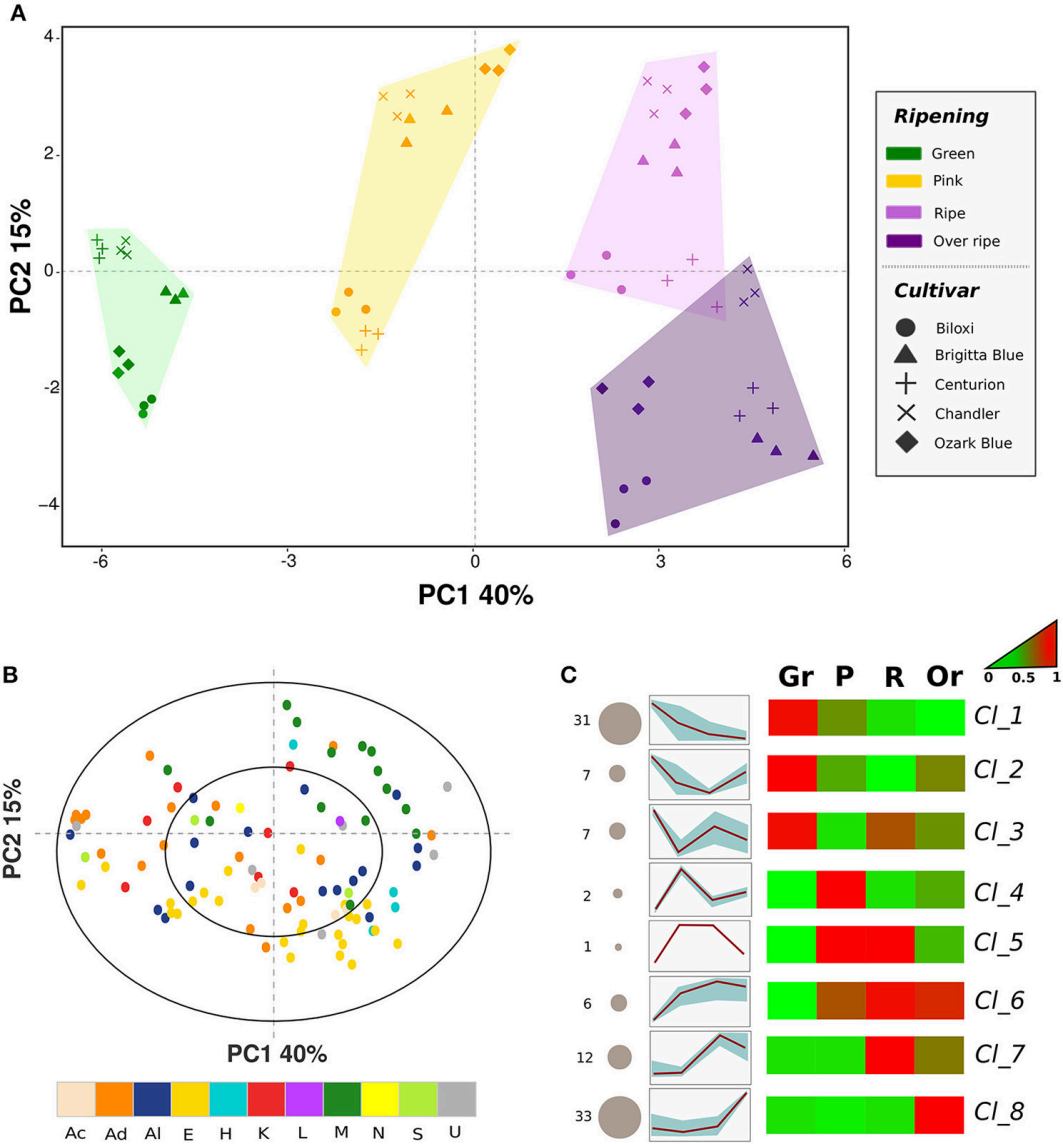
**Multivariate analysis of the blueberry VOC profile assessed by SPME-GC-MS**. Plot **(A)** depicts the VOC profile distribution of five blueberry cultivars at four ripening stages over the PCA score plot defined by the first two principal components. Plot **(B)** shows the projection of the VOCs identified by SPME-GC-MS analysis. Each compound is reported using different color according to the chemical class [acids (Ac), aldehydes (Ad), alcohols (Al), esters (E), hydrocarbons (H), ketones (K), lactones (L), monoterpenes (M), norisoprenoids (N), sesquiterpenes (S), unknowns (U)]. Plot **(C)** reports the sorting of all compounds into eight significant clusters defined by SOTA (Self-organizing tree algorithm) analysis. Several additional information are reported next to each SOTA cluster: the number of compounds (number plus size of circle), heatmap and plot of compound fold changes among time series (normalized data to 0–1 range). Details about each SOTA cluster are reported into Supplementary Table 1.

Principal component analysis (PCA) was carried out to describe relations among blueberry cultivars, ripening stages, and VOCs (Figure 2A). Sixty-five percent of the total variability was accounted for the first three principal components. Differences related to fruit ripening stages were mostly explainable by the first principal component (PC1: 40%), while differences between cultivars were better defined using the second component (PC2: 15%). Moreover, the variability described by the second principal component was essential to distinguish fruit belonging to the “ripe” or “overripe” classes. Overripe blueberries were indeed differentiated from ripe ones by more negative values of PC2, without any significant variations of PC1. This variability concurred with an increased concentration of compounds already present at significant levels in ripe fruits (such as ethyl benzene, xilene, hexanol, or 1-octen-3-ol), and, mostly, with the synthesis of compounds not detectable (or detectable only at trace levels) in ripe fruits that were for the most esters (i.e., ethyl acetate, methyl 3-methylbutanoate, or ethyl 3-methylbutanoate; loading plot Figure 2B). In addition, overripe fruits were characterized by a lower concentration of monoterpenes (i.e., limonene, linalool, α-terpineol, and β-myrcene) that were fully synthesized in ripe fruit.

PCA analysis indicates that VOC emission of fruit at green maturity stages was characterized by high levels of aldehydes (i.e., 3-hexenal, 2-hexenal, 2-pentenal, or 2-heptenal) and of alcohols derived by these aldehydes, for instance (Z)-3- hexen-1-ol and 3-methyl butanol. The aromatic profile of unripe fruit was completed by some hexenyl esters (i.e., 3-hexenyl acetate, 3-hexenyl butanoate), one aromatic ester (ethyl benzoate), several ketones (i.e., 2-heptenone and 2-nonanone), one monoterpene (1,8-cineole), and by the sesquiterpenes (E)-caryophyllene and caryophyllene oxide.

The aromatic profile of fruit harvested at the pink ripening stage was mostly defined by both the aforementioned compounds (for the most aldehydes) detected in greens stage blueberries, which concentrations decreased at this ripening stage. Moreover, some monoterpenes, such as linalool, linalool oxide, 4-terpineol and geranyl acetate were produced at this ripening stage.

All VOCs detected in this study, beyond the biochemical classification, were grouped based on their concentration fold changes disclosed during the entire ripening process. All compounds were significantly sorted into eight clusters defined by SOTA (Self-organizing tree algorithm) analysis (Figure 2C, Table 1). Indeed, SOTA algorithm allowed a clustering based on relative fold changes of a compound among time series. The two groups with the higher number of VOCs were “cluster_1” and “cluster_8” respectively represented by 31 and 33 compounds. VOCs belonging to “cluster_1” were mostly produced by green blueberries and they were suddenly reduced during fruit ripening. Oppositely, compounds of “cluster_8” were exclusively synthesized in the last ripening step in overripe fruits. All left compounds were divided into the remaining six clusters (from “cluster_2” to “cluster_7”), which showed different dynamics patterns of VOCs concentration fold changes from green to over ripe.

### Direct injection VOC profiling by PTR-ToF-MS

Fruit samples analyzed by PTR-ToF-MS were prepared similarly to the ones used for SPME-GC-MS analysis in order to compare these two methodologies. The PTR-ToF-MS setting adopted in this study allowed the detection of the full VOC spectra in 1 s. Only the first 30 s of the full measurement (120 s) were analyzed and averaged, in order to avoid possible measurement inaccuracies caused by an excessive dilution of the sample headspace. The whole VOC spectra, assessed in triplicate for samples, were reduced from 293 to 105 masses, applying noise, and correlation coefficient thresholds (Table 2).

**Table 2:** **Volatile compounds detected by PTR-ToF-MS in blueberry fruits at different ripening stages**.

***m/z***	**Formula**	**SOTA^a^**	**Tentative identification**	**Min^b^**	**Max^b^**	**Mean^b^**
28.008		1	n.i.	0.6	0.7	0.6
29.040	C_2_${\text{H}}_{5}^{+}$	8	Ethanol fragment	1.9	64.8	2.4
30.995		1	n.i.	1.0	1.2	1.0
31.019	CH_3_O^+^	8	Formaldehyde	2.8	14.2	5.2
33.994	O[18]O^+^	1	n.i.	4.7	5.1	4.9
34.037	[13]CH_4_OH^+^	8	Methanol	3.4	92.7	17.8
39.023	C_3_${\text{H}}_{3}^{+}$	3	Common fragment	5.2	29.8	12.2
41.039	C_3_${\text{H}}_{5}^{+}$	3	Common fragment	12.3	56.3	21.9
43.018	C_2_H_3_O^+^	3	Common fragment	26.1	129.8	50.3
43.054	C_3_${\text{H}}_{7}^{+}$	3	Common fragment	3.3	23.0	8.8
45.033	C_2_H_4_OH^+^	8	Acetaldehyde	59.8	1358.0	176.1
45.992	${\text{NO}}_{2}^{+}$	1	n.i.	1.2	1.6	1.4
47.013	CH_3_O${\text{O}}_{2}^{+}$	5	Formic acid	7.2	18.3	10.2
47.024		1	n.i.	4.4	5.2	4.7
47.049	C_2_H_6_OH^+^	8	Ethanol	2.3	295.5	4.5
49.012	CH_4_SH^+^	2	Methanethiol	0.2	0.8	0.4
51.023		6	n.i.	0.2	0.9	0.4
51.043	CH_3_OH^^*^^H_3_O^+^	8	Methanol cluster	4.7	132.4	25.0
53.039	C_4_${\text{H}}_{5}^{+}$	3	n.i.	1.5	8.9	3.9
55.018		2	n.i.	0.2	1.8	0.7
55.054	C_4_${\text{H}}_{7}^{+}$	2	Common fragment	21.4	193.8	81.5
55.934		1	n.i.	1.1	1.2	1.2
57.033	C_3_H_4_OH^+^	5	Common fragment	48.1	779.2	231.2
57.070	C_4_${\text{H}}_{9}^{+}$	3	1-Octanol^*^, high alcohol fragment	0.1	5.1	3.6
59.049	C_3_H_6_OH^+^	7	Acetone	26.6	880.1	37.6
61.028	C_2_H_4_O_2_H^+^	6	Acetic acid, common ester fragment	10.2	52.5	19.0
63.026	C_2_H_6_SH^+^	4	Dimethyl sulfide, Ethanethiol	0.8	42.7	1.6
63.043	C_2_H_4_O^^*^^H_3_O^+^	8	Ethanol cluster	0.2	3.5	0.4
65.022		4	n.i.	0.2	2.1	0.3
65.039	C_5_${\text{H}}_{5}^{+}$	3	n.i.	0.1	0.4	0.1
67.054	C_5_${\text{H}}_{7}^{+}$	3	n.i.	0.9	4.2	1.8
69.034	C_4_H_4_OH^+^	2	Furan	0.3	1.1	0.6
69.070	C_5_${\text{H}}_{9}^{+}$	3	Aldehyde fragment	3.8	22.4	8.7
70.039		3	n.i.	0.0	0.3	0.1
71.049	C_4_H_6_OH^+^	2	Butenal	1.6	6.6	3.6
71.086	C_5_${\text{H}}_{11}^{+}$	4	3-Methyl-1-butanol^*^, 2-Methyl-1-butanol^*^, Pentanol^*^	1.1	10.8	2.7
73.028	C_3_H_4_O_2_H^+^	1	n.i.	0.8	1.0	0.9
73.048		1	n.i.	0.7	1.2	1.0
73.065	C_4_H_8_OH^+^	2	Butanale, Isobutyraldehyde	2.5	7.2	4.3
75.027	C_3_H_6_SH^+^	1	Allyl mercaptan, 3-mercaptopropanol	1.1	1.7	1.4
75.044	C_3_H_6_O_2_H+	8	Methyl acetate^*^	0.9	45.4	1.2
78.047	C_6_${\text{H}}_{6}^{+}$	1	n.i.	2.4	2.6	2.5
79.055	C_6_${\text{H}}_{7}^{+}$	3	Benzene	4.0	11.6	7.9
80.060	C_5_[13]${\text{CH}}_{7}^{+}$	3	n.i.	0.4	2.0	0.8
81.070	C_6_${\text{H}}_{9}^{+}$	4	Fragment of aldehydes (hexenals); fragment of terpenes (linalool)	19.1	1497.7	283.8
83.049	C_5_H_6_OH^+^	2	Methylfuran	0.5	2.9	1.3
83.086	C_6_${\text{H}}_{11}^{+}$	5	(E)-3-hexen-1-ol^*^, (Z)-3-hexen-1-ol^*^, (Z)-2-hexen-1-ol^*^, Hexanal^*^,2-Hexanone	14.3	123.0	49.9
85.065	C_5_H_8_OH^+^	4	3-Penten-2-one	2.2	17.5	6.7
85.100	C_6_${\text{H}}_{13}^{+}$	6	Hexanol^*^	0.3	1.2	0.4
87.044	C_4_H_6_O_2_H^+^	3	Butyrolactone^*^	0.8	4.5	1.1
87.080	C_5_H_10_OH^+^	3	2-Methyl butanal^*^, 3-Methyl butanal^*^	1.0	4.6	1.8
89.060	C_4_H_8_O_2_H^+^	8	Ethyl acetate^*^	0.8	11.2	1.2
90.949		1	n.i.	1.8	1.9	1.9
91.057	C_7_${\text{H}}_{7}^{+}$	1	Benzyl fragment^*^	0.9	1.5	1.1
93.037	C_3_H_8_OSH^+^	1	2-(Methylthio)ethanol	1.4	1.7	1.5
93.070	C_7_${\text{H}}_{9}^{+}$	6	Monoterpene fragment	0.5	2.1	0.8
94.041		1	n.i.	0.4	0.5	0.4
95.022		1	n.i.	0.4	0.6	0.5
95.049	C_6_H_6_OH^+^	1	Phenol	1.6	2.2	1.9
95.086	C_7_${\text{H}}_{11}^{+}$	1	(E)-2-heptenal^*^, Monoterpene fragment	1.1	4.2	2.2
97.065	C_6_H_8_OH^+^	4	(E,Z)-2,4-hexadienal^*^, (E,E)-2,4-hexadienal^*^	0.4	3.1	1.2
97.102	C_7_${\text{H}}_{13}^{+}$	3	Heptanal^*^, fragment	0.5	2.5	0.6
99.080	C_6_H_10_OH^+^	3	(Z)-3-hexenal^*^, (lZ)-2-hexenal^*^, (E)-2-hexenal^*^	17.9	433.9	145.8
101.064	C_5_H_8_O_2_H^+^	3	2,3-Pentanedione	0.3	0.8	0.4
101.095	C_6_H_12_OH^+^	5	Hexanal^*^	2.1	16.9	6.3
103.076	C_5_H_10_O_2_H^+^	8	Ethyl propanoate^*^	0.5	8.0	0.6
105.071	C_8_${\text{H}}_{9}^{+}$	6	Phenethyl alcohol^*^, Styrene	0.1	0.4	0.2
105.938		1	n.i.	0.2	0.2	0.2
107.050	C_7_H_6_OH^+^	2	Benzaldehyde^*^	0.2	0.5	0.3
107.086	C_8_H_10_H^+^	1	Ethyl benzene^*^, p-Xylene^*^, m-Xylene^*^	3.7	15.6	9.6
107.953		1	n.i.	0.3	0.3	0.3
108.957		1	n.i.	0.7	0.8	0.7
109.102	C_8_${\text{H}}_{13}^{+}$	5	n.i.	2.1	7.7	3.3
111.081	C_7_H_10_OH^+^	3	(E,E)-2,4-heptadienal^*^	0.3	1.1	0.6
111.118	C_8_${\text{H}}_{15}^{+}$	1	(E)-2-Octenal^*^, Octanal^*^,1-Octen-3-ol	0.3	0.5	0.4
113.027		2	n.i.	0.1	0.2	0.2
113.060	C_6_H_8_O_2_H^+^	4	Sorbic acid	0.2	2.0	0.4
113.097	C_7_H_12_OH^+^	1	(E)-2-heptenal^*^	0.3	1.2	0.6
115.077	C_6_H_10_O_2_H^+^	2	Ethyl (2E)-2-butenoate^*^	0.2	0.6	0.3
115.113	C_7_H_14_OH^+^	4	2-Heptanone^*^, Heptanal	0.2	24.2	1.3
117.092	C_6_H_12_O_2_H^+^	8	Ethyl isobutanoate^*^, Methyl-2-methyl butanoate^*^, Methyl isovalerate^*^, Ethyl butyrate^*^, Hexanoic acid^*^	0.9	17.6	1.2
119.088	C_9_${\text{H}}_{11}^{+}$	1	3-Phenylpropanol	0.5	0.6	0.5
121.066	C_8_H_8_OH^+^	2	Acetophenone^*^, Phenylacetaldehyde	0.6	1.6	0.9
121.103	C_9_${\text{H}}_{13}^{+}$	1	1,3,5-Trimethylbenzene	0.2	0.3	0.3
123.118	C_9_${\text{H}}_{15}^{+}$	1	2-Nonenal^*^, (E)-2-nonenal^*^	0.3	0.4	0.3
125.097	C_8_H_12_OH^+^	3	6-Methyl-3,5-heptadien-2-one	0.2	0.6	0.3
126.903		1	n.i.	0.3	0.3	0.3
127.113	C_8_H_14_OH^+^	5	1-Octen-3-one^*^, 6-Methyl-5-hepten-2-one^*^, (E)-2-octenal^*^, β-Myrcene^*^, Limonene^*^, (E)-β-Ocimene^*^, α-Terpinolene^*^	0.8	3.0	1.4
129.128	C_8_H_16_OH^+^	1	2-Octanone^*^, Octanal^*^, 1-Octen-3-ol	0.2	0.5	0.3
131.107	C_7_H_14_O_2_H^+^	8	Ethyl-2-methyl butanoate^*^, Ethyl isovalerate^*^	0.2	6.0	0.3
133.102	C_10_${\text{H}}_{13}^{+}$	1	Thymol	0.1	0.4	0.2
135.118	C_10_${\text{H}}_{15}^{+}$	7	HO-trienol^*^	0.3	3.6	0.7
137.134	C_10_${\text{H}}_{17}^{+}$	3	1,8-cineole^*^, Linalool^*^, 4-Terpineol^*^, α-Terpineol^*^, Nerol^*^, Geraniol^*^	1.6	13.2	5.8
139.076	C_8_H_10_O_2_H^+^	1	5,5-Dimethyl-2-cyclohexen-1,4-dione	0.1	0.3	0.2
139.115	C_9_H_14_OH^+^	2	n.i.	0.2	0.4	0.3
141.129	C_9_H_16_OH^+^	1	2-Nonenal^*^, (E)-2-nonenal^*^, Ethyl sorbate	0.2	0.3	0.3
143.108	C_8_H_14_O_2_H^+^	3	(Z)-3-hexenyl acetate^*^, 2-Hexenyl acetate^*^	0.2	0.6	0.3
143.145	C_9_H_18_OH^+^	3	2-Nonanone^*^, Nonanal	0.1	0.8	0.4
144.914		1	n.i.	0.1	0.2	0.2
145.124	C_8_H_16_O_2_H^+^	1	Ethyl hexanoate^*^, Hexyl acetate^*^, Octanoic acid^*^	0.5	0.8	0.7
153.129	C_10_H_16_OH^+^	7	HO-trienol^*^, 2,4-Decadienal	0.3	6.0	0.9
155.144	C_10_H_18_OH^+^	5	1,8-Cineole^*^, Linalool^*^, 4-Terpineol^*^, α Terpineol^*^, Nerol^*^, Geraniol^*^	0.2	0.5	0.4
159.140	C_9_H_18_O_2_H^+^	1	Nonanoic acid^*^	0.7	1.6	1.2
173.156	C_10_H_20_O_2_H^+^	1	Decanoic acid^*^	0.4	0.8	0.7
177.166	C_13_${\text{H}}_{21}^{+}$	1	Geranyl acetone^*^	0.2	0.4	0.3

a *SOTA (self-organizing tree algorithm) clusters based on Figure 3*.

b *ppb_v_*.

* *Compound detected also by SPME-GC-MS analysis*.

PCA analysis (Figure 3A) was carried out to describe the blueberry VOC profile regarding cultivars (“Biloxi,” “Brigitta Blue,” “Centurion,” “Chandler,” and “Ozark Blue”) and ripening stages (green, pink, ripe, overripe). Seventy-two percent of the total variation was accounted for the first three principal components. Similarly to SPME-GC-MS analysis, differences between fruit sampled at different ripening stages were mostly explainable by the first principal component (PC1: 43%), while the second component (PC2: 18%) mostly defined differences between cultivars. Furthermore, PC2 variability allowed the separation of overripe fruit from ripe ones: overripe fruits, besides the cultivar “Chandler,” were all displaced into the PCA quadrant determined by positive values of PC1 and negative ones of PC2. Based on the loading plot (Figure 3B), this discrimination was mostly explainable by masses related to alcohols such as *m/z* 29.040 and 47.049 (ethanol), *m/z* 34.037 (methanol isotope; the nominal mass of methanol, *m/z* 33.030, was not considered in this study because in some samples its concentration was above the maximum threshold of accuracy), acetone (*m/z* 59.048), acetaldehyde (*m/z* 45.033), formaldehyde (*m/z* 31.019), and several masses tentatively associated to esters such as *m/z* 61.028, 75.044, 89.060, 103.076, 117.092, 131.107.

**Figure 3:**
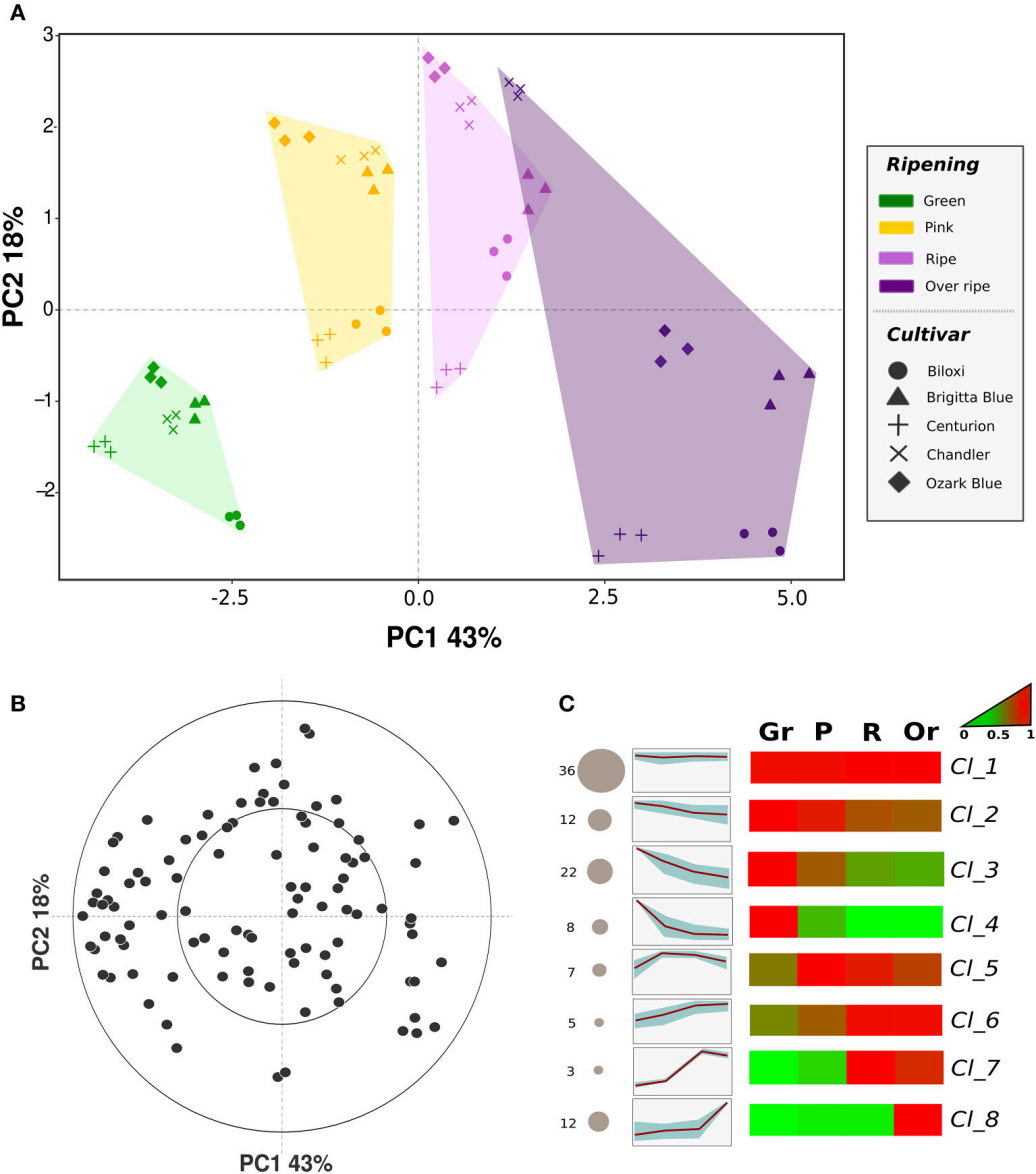
**Multivariate analysis of the blueberry VOC profile assessed by PTR-ToF-MS**. Plot **(A)** depicts the VOC profile distribution of five blueberry cultivars at four ripening stages over the PCA score plot defined by the first two principal components. Plot **(B)** shows the projection of the VOCs identified by PTR-ToF-MS analysis (the high resolution vector form of the loading plot is illustrated in Supplementary Figure 4). Plot **(C)** reports the sorting of all compounds into eight significant clusters defined by SOTA (Self-organizing tree algorithm) analysis. Several additional information are reported next to each SOTA cluster: the number of compounds (number plus size of circle), heatmap and plot of compound fold changes among time series (normalized data to 0–1 range). Details about each SOTA cluster are reported into Supplementary Table 2.

The aroma profile of unripe fruits, especially of the green ones, was mostly defined by negative values of PC1 that means a higher concentration of masses related to aldehydes, such as *m/z* 99.080, 81.070, 69.070, or 43.019, esters with “green” fragrances such as hexadienal and hexenyl acetate (*m/z* 97.06 and 143.108), butyrolactone (*m/z* 87.044), and sulfuric compounds (*m/z* 63.026 and 93.037, tentatively identified as dimethyl sulfide and 2-(methylthio)ethanol).

Almost one third of the PTR-ToF-MS masses was not strongly correlated (*R* < 0.5) with any of the three principal components. These masses, indeed, were detectable only at low concentrations (average level lower than 2 ppbv) and/or they did not vary significantly between cultivars and ripening stages such as for *m/z* 107.08 (ethyl benzene and/or xylene) and *m/z* 95.086 ((E)-2-heptenal).

As for SPME-GC-MS analysis, all 105 masses detected by PTR-ToF-MS were grouped into eight significant SOTA clusters based on their concentration fold changes during the entire ripening process (Figure 3C, Table 2). As formerly revealed by the PCA analysis, “cluster_1” grouped a set of 36 masses that did not significantly vary between cultivars and ripening stages. “Clusters_2, _3, and _4” sorted VOCs that were mostly produced by unripe fruits and they diminished during fruit ripening. Each of the three clusters was characterized by a different depletion slope of VOCs concentration. The remaining VOCs, that were mostly produced during fruit ripening, were arranged into the remaining four clusters, namely “cluster_5, _6, _7, and _8.”

“Cluster_5” was composed by seven VOCs masses whose concentration was highest in fruits assessed at the pink ripe stage. This concentration remained stable, or it slightly diminished, during the last ripening phases (ripe and overripe). The main masses grouped into this cluster were: *m/z* 47.013 (formic acid); *m/z* 83.086 (hexenols and/or hexenal fragment); *m/z* 101.09 (hexenal); *m/z* 127.113 (6-methyl-5-heptenone and terpenes such as myrcene and limonene); *m/z* 155.144 (terpenes such as linalool, geraniol, or cineole).

“Cluster_6” gathered five VOCs masses characterized by a constant and almost linear concentration increment during fruit ripening. Some of these masses were *m/z* 61.028 (acetic acid and common ester fragment), *m/z* 85.100 (hexanol), *m/z* 93.07 (monoterpene fragment), and *m/z* 105.071 (phenethyl alcohol).

“Cluster_7” was only composed by three VOC masses namely *m/z* 59.049 (acetone), *m/z* 135.118 and 153.129 (HO-trienol). These VOCs were mostly produced by fruit analyzed at ripe stage.

“Cluster_8,” lastly, counted 12 VOCs masses whose concentration stood at low basal levels till the ripe stage and it greatly rose in overripe fruit. Among these compounds there were masses related to ethanol (*m/z* 29.040, 47.049, and 63.043), to methanol (*m/z* 34.037 and 51.043) and to ester compounds (*m/z* 75.044, 89.060, 103.076, 117.092, and 131.107).

**Figure 4:**
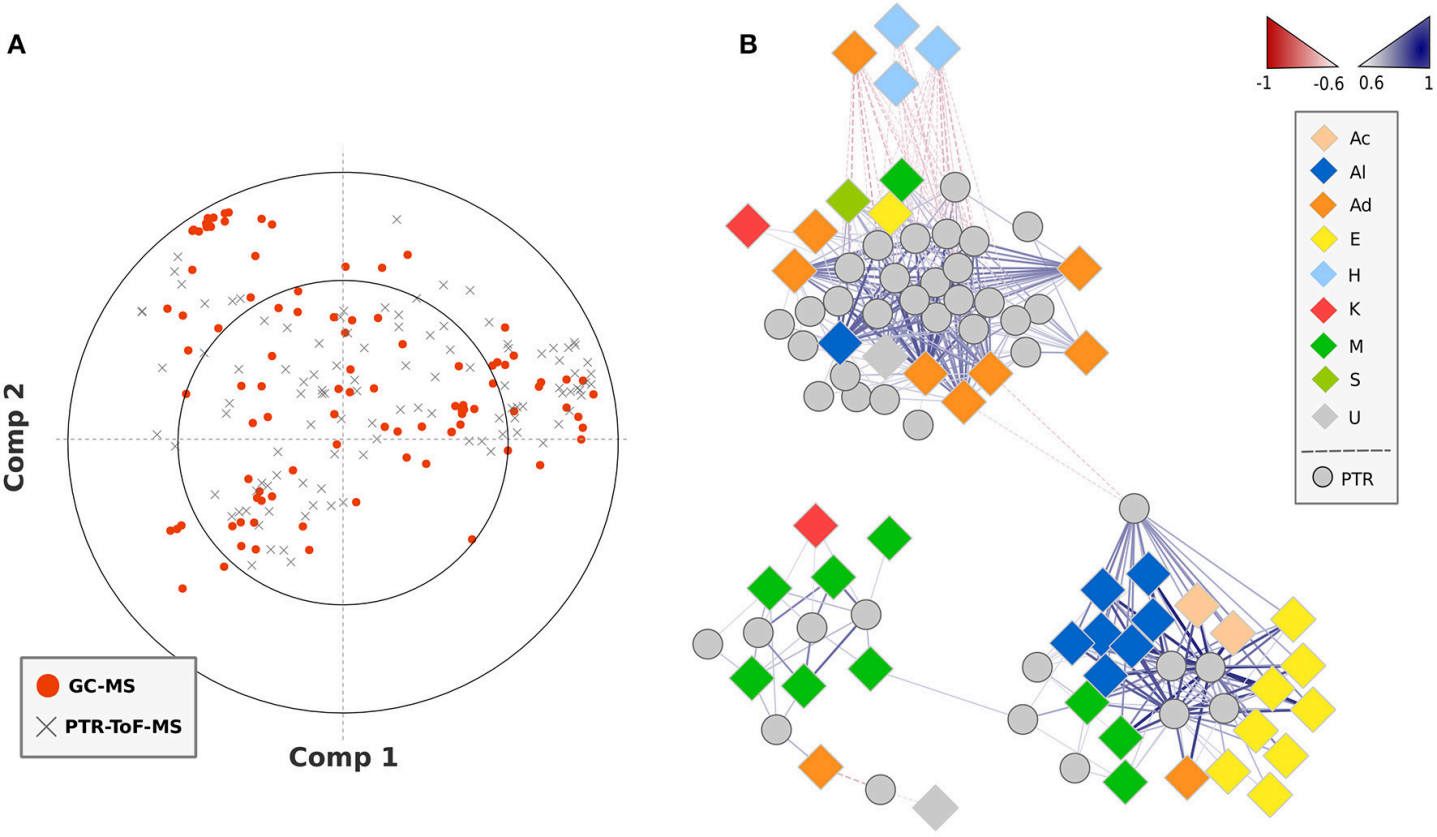
**PLS regression of VOC data obtained by SPME-GC-MS and PTR-ToF-MS analysis**. Plot **(A)** reports the loading plot of the PLS regressions analysis assessed over the SPME-GC-MS and PTR-ToF-MS data. The plot **(B)** disclosed the correlation analysis network (CAN) built on significant (*p* < 0.01) PLS correlations between VOCs detected by SPME-GC-MS and masses quantified by PTR-ToF-MS. The gradient color coding of the edges, as well as the line thickness, denotes the level of correlation (0.6–1). Positive and negative correlations are shown by blue and red gradient color. The high resolution vector form of the correlation network is illustrated in Supplementary Figure 5.

A PLS regression network was created combining VOC data obtained by SPME-GC-MS and PTR-ToF-MS analysis (Figure 4). Based on the obtained regressions, graphically reported in the loading plot of Figure 4A, the network was built on significant (*p* < 0.01) correlations between VOCs detected by SPME-GC-MS and masses quantified by PTR-ToF-MS only taking into account correlation values >0.6 or <–0.6. The gradient color coding of the edges (blue and red gradient color), as well as the line thickness, denotes the level of correlation (0.6–1). Most of the PTR-ToF-MS peaks considered in this study were putatively identified (Table 2) based on PLS regression analysis, *in silico* fragmentation of the compounds previously detected by SPME-GC-MS, and fragmentation analysis of commercial standards. Indeed, lots of incorrect attributions would be obtained by merely considering the SPLS results. For instance *m/z* 89.06 was highly correlated (*R* > 0.9) with at least eight VOCs detected by SPME-GC-MS, such as the esters methyl-2-methylbutanoate or 3 methyl-3-hydroxy-3-methylbutanoate and the alcohols 1-octanol or 1-nonanol, even though all these molecules were differently fragmented during protonization. Therefore, ethyl acetate resulted to be the only detected compound by SPME-GC-MS highly correlated with *m/z* 89.060 and with an accurate chemical fragmentation.

#### VOCs characterization of vaccinium species by PTR-ToF-MS

**Figure 5:**
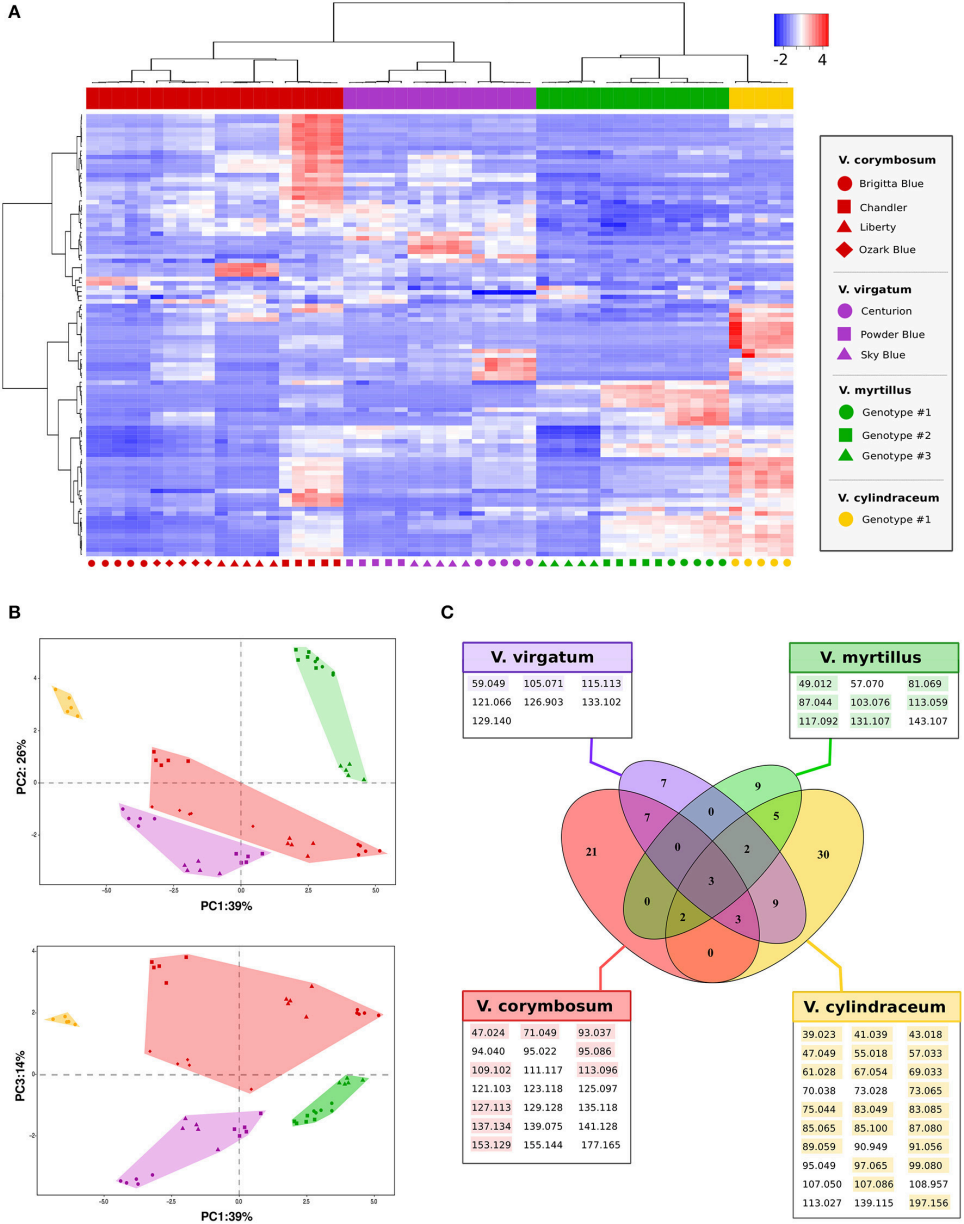
**Multivariate VOC characterization of ***Vaccinium*** species by PTR-ToF-MS**. Plot **(A)** represents the heat map and two dimensional hierarchical dendrograms of VOC assessed in 11 *Vaccinium* in five biological replicates. Cluster analysis was performed using Ward's method on centered and scaled data. *Vaccinium* accessions are grouped and clustered by columns, while VOCs are organized by rows. Plot **(B)** depicts the VOC profile distribution of the *Vaccinium* accessions over the PCA score plot defined by the first three principal components (loading plots of the PCA analysis are reported into Supplementary Figure 6). Symbols and colors refer, respectively, to the accession names and to the *Vaccinium* species reported in plot **(A)**. In the Venn diagram, plot **(C)**, the VOCs significantly (*p* < 0.01) more detectable in each *Vaccinium* species are grouped together (detailed ANOVA results are reported into Supplementary Table 3). For each species the significant *m/z* detected with a concentration higher than 2 ppbv are colourly highlighted.

In addition to the previous study, PTR-ToF-MS methodology was applied to identify the VOCs profiles of different blueberry species assessed at the full ripe stage. In detail, we analyzed four cultivars of *V. corymbosum* L. (“Brigitta Blue,” “Chandler,” “Liberty,” and “Ozark Blue”), three cultivars of *V. virgatum* Aiton (“Centurion,” “Powder Blue,” and “Sky Blue”), three ecotypes of *V. myrtillus* L. propagated from different mountain locations of Trentino, and one accession of *V. cylindraceum* Smith. The entire volatile profiles assessed for these 11 accessions, in five biological replicates, were organized and depicted by the heat map showed in Figure 5A, using a data-set reduced to 98 masses by applying noise and correlation coefficient thresholds (all data are “centered” and “scaled”). The vertical dendrogram of the heat map shows the grouping of PTR-ToF-MS masses based on their relative abundance. On the same heat map all blueberry accessions were organized by a hierarchical clustering, based on VOCs relative content. The hierarchical clustering revealed a significant grouping of the *Vaccinium* accessions based on their taxonomic differences, since each of the four species was grouped into a clearly separate cluster.

This separation was also confirmed by the PCA analysis done on Log-transformed data (Figure 5B). Seventy-nine percent of the total variability among aroma profile was accounted for the first three principal components (PC1: 39%; PC2: 26%; PC3: 14%). Based on these PCA results, all cultivars were properly and distinctly distributed based on their aromatic profile confirming the defined clusterization among *Vaccinium* species and low variability among biological replicates. For instance, the aromatic profile of *V. myrtillus* L. and *V. cylindraceum* Smith accessions were significantly distinct mostly based on PC1 and PC3-values. According to the PCA loading plot (Supplementary Figure 4) this separation was mostly explainable by a higher concentration of most of detected compounds (negative values of PC1). *V. corymbosum* L. and *V. virgatum* Aiton, instead, mostly differed according to PC3-values. However, the variability among these last two species was broad, with cultivars characterized by an intense VOC profile, such as “Chandler” or “Centurion,” and others with reduced VOC concentrations, such as “Brigitta Blue.”

All these indicative differences in aroma composition detected by PTR-ToF-MS are summarized into the Venn diagram (Figure 5C) where the VOCs significantly (*p* < 0.01) more detectable in each *Vaccinium* species are grouped together (Supplementary Table 1). Among the four species assessed in this study, *V. cylindraceum* L. was the most aromatic one. Its aroma profile was defined by 30 masses that were measured at significantly higher concentration such as some alcohols (*m/z* 47.049 and 85.100), ester fragments (i.e., *m/z* 61.028, 75.044, and 89.059), aldehydes (*m/z* 83.085, 97.065, and 99.080), and aromatic hydrocarbon compounds (*m/z* 95.049 and 107.086). The *V. myrtillus* L. ecotypes were characterized by a less intense VOC profile than *V. cylindraceum* L. This profile, on average levels, was defined by an increased amount of *m/z* 81.070 (C6 aldehyde and terpene fragment) and some ester related masses (i.e., *m/z* 103.076, 117.092, 131.107, and 143.108). The four cultivars of *V. corymbosum* L. differed from the other accessions for 21 masses. However, these VOCs were detectable at lower concentration, since only nine of them revealed at concentration higher than 2 ppbv, such as some terpene related masses (i.e., *m/z* 93.037, 127.113, or 137.135). Lastly, the VOCs profile of the cultivars of *V. virgatum* Aiton revealed several masses detected at the same level of *V. corymbosum* L. (13 masses) and *V. cylindraceum* Smith (11 masses), and 7 masses significantly more produced with only three of them with concentration higher than 2 ppb_*v*_ (*m/z* 59.049, 105.071, and 115.113).

## Discussion

With an increased consumption of fresh blueberries in the last decade, a whole new generation of cultivars has to be released and bred, at least in part, for improved fruit quality, shelf life stability, and extension of the fresh-market harvest season (Gilbert et al., 2014). Although flavor is a complex trait, relatively simple measurements are commonly used in an attempt to quantify flavor differences, such as titratable acidity, TTS, and firmness. Nevertheless, identification of VOCs that correspond to the fruity, intense, sweet, and characteristic blueberry flavors could help breeders to select for cultivars with a more desirable flavor. This is an essential first step in growing demand for fruits and vegetables rather than merely maintaining existing markets (Folta and Klee, 2016).

However, flavor phenotyping is expensive, subject to environmental variation, not amenable to high-throughput assays, and beyond the means of most breeding programs. Given the importance of aroma to define the complexity of flavor and consumer preferences, the development of techniques to rapidly, accurately, and comprehensively assess VOCs is crucial. This strategy may prevent the unintended negative consequences of breeding on other quality traits of blueberry, as it already happened in several important fruit species, such as strawberry, apple, peach, and tomato (Goff and Klee, 2006; Klee, 2010; Rambla et al., 2014; Folta and Klee, 2016; Farneti et al., 2017) where the breeding pressure led to an evident aroma decline.

Volatile compounds, as the majority of secondary plant metabolites, are detectable in blueberry fruits with high variability according to genetic and environmental differences (Gilbert et al., 2015) and, above all, to the biological ripening stage of the fruit at the time of analysis (Gilbert et al., 2013). In this survey, the environmental variable effect was fully reduced by using plants grown in the same experimental field with identical agronomic practices. The two main variance factors introduced in this experimental design were the genetic variability (five cultivars of *V. corymbosum* L. and *V. virgatum* Aiton; 11 accessions of *V. corymbosum* L., *V. virgatum* Aiton, *V. cylindraceum* Smith, and *V. myrtillus* L.) and the harvest ripening stages (green, pink, ripe, and overripe), that were analytically defined based on color, pH, titratable acidity, TSS, and texture.

This comprehensive characterization of blueberry aroma, assessed by chromatographic (SPME-GC-MS) and direct injection (PTR-ToF-MS) spectrometric techniques, allowed the identification of most compounds that may affect blueberry quality. To date, this is the most detailed characterization of blueberry aroma with 106 compounds, detected and tentatively identified by gas-chromatographic analysis. Moreover, PTR-ToF-MS analysis resulted to be complementary to SPME-GC-MS since, over the 105 significant masses detected, several compounds, in this study, were only detected and quantified by PTR-ToF-MS analysis such as methanol, ethanol, acetaldehyde, acetone, acetic acid, or some sulfuric compounds (C_2_H_6_SH^+^, *m/z* 63.026).

All VOCs detected in this study with both chromatographic and direct injection techniques, beyond the biochemical classification, were grouped based on their concentration fold changes during the entire ripening process. This physiological classification straightly unravels how complex the blueberry VOCs profile can be, and that should not be simplified as the interaction of <10 compounds (i.e., linalool, trans-2-hexenol, trans-2-hexenal, or hexanal; (Parliment and Kolor, 1975; Hirvi and Honkanen, 1983; Du et al., 2011). Based on the SOTA classification, indeed, all compounds can be broadly gathered into three main groups: (i) VOCs that do not significantly vary between cultivars and ripening stages and that are detectable only at low concentrations; (ii) VOCs that are mostly synthesized by unripe (green) blueberries and that are reduced during fruit ripening; (iii) VOCs that are exclusively synthesized in the last ripening steps (ripe and/or overripe). The last two groups, in addition, can be further divided into sub-clusters characterized by different depletion/production slopes of VOCs concentrations. Most of the compounds that are commonly considered being responsible for blueberry aroma are synthesized by the fruit in the ripe stage, such as linalool and majority of monoterpenes, (Z)-2-hexen-1-ol, and hexanal, or they are mostly detected in fruits at the pink stage of ripening, such as (E)-2-hexenal. (E)-2-hexenol has been linked to green-viney, sweet, and pungent characters, while (E)-2-hexenal has been described as fresh, leafy green, floral, sweet, and pungent (Hongsoongnern and Chambers, 2008). Linalool has often been cited as characteristic of blueberry aroma and mostly it is associated with a floral, fruity, citrus flavor (Parliment and Kolor, 1975; Hirvi and Honkanen, 1983; Du et al., 2011). This enhanced synthesis of terpenes during fruit ripening suggests the feasible upregulation of genes involved into the mevalonate and methylerythritol pathways and of specific terpene synthases, such as linalool synthase (Nagegowda, 2010).

However, it is not necessarily the total amount of the volatiles synthesized in each fruit that is important to flavor, but the presence of specific volatiles, sometimes even in small amounts, with low odor thresholds (Tieman et al., 2012; Folta and Klee, 2016). Esters, although being present in lower average concentration compared to the aforementioned compounds, were important to fully decipher the blueberry aroma, especially for their “sweet” and “fruity” fragrances (Du and Rouseff, 2014). A large fraction of these esters, such as ethyl acetate, methyl isovalerate, ethyl isovalerate, methyl 2-methylbutanoate, are exclusively synthesized in the last phase of ripening and magnified in overripe fruit (SOTA cluter_8 for both PTR-ToF-MS and SPME-GC-MS data). To date esters are not considered as important as aldehydes or terpenes to fully decipher the blueberry aroma, mostly because the majority of studies aimed to correlate sensory consumer perception and VOCs did not consider fruits at foremost ripening stage such as over ripe fruit or fruit after a long storage period. In our opinion these VOCs, mostly synthesized at the full ripening stage of the fruit, have to be considered crucial to fully decipher the aroma profile due to their ecological/evolutionary role to attract eaters. This is a common “ecological-strategy” of climacteric fruit, such as apple, melon, tomato, peach, in which the VOCs synthesis coincides with the ripening and the evolution of attractive quality attributes such as the sugar/acid ratio (Goff and Klee, 2006). On the other hand compounds that are present in green fruit, and that are drastically reduced during ripening (i.e., 1,8-cineole, (Z)-3-hexenal, (E,E)-2,4-hexadienal, (Z)-3-hexenyl acetate, or (E)-caryophyllene) may be considered as “not attractive” or even as “repellent” and so they might not be so important to enhance the final fruit quality perceived by consumer. Notably, the content evolution of 1,8-cineole during fruit ripening reveals a totally contrasting behavior than most of blueberry terpenes, especially linalool. The extremely high concentration assessed in unripe blueberries suggets the upregulation of genes involved in the conversion of gernayl diphosphate into 1,8-cineole, such as terpineol synthase and 1,8-cineole synthase (Piechulla et al., 2016).

VOCs are not only responsible for the blueberry flavor, they also interact in the ecological network between plant/fruit and the environment and respond to stress conditions (e.g., herbivore or pathogen attack). Furthermore, it is important to know the dynamics of VOCs production not only for quality issues but also to predict the resistance of the fruit to abiotic and biotic stress. Several insects, for instance, are attracted by VOCs emitted by fruit such as *Drosophila suzukii* (Scheidler et al., 2015) or *Rhagoletis mendax* (Lugemwa et al., 1989). Identification of these compounds, often present at trace concentrations, would be an essential component of elucidating the mechanisms of oviposition site selection by these insects and also a helpful tool for breeding activities focused on the development on more resistant accessions.

The obtained results demonstrated the complementarity between chromatographic and direct-injection spectrometric techniques to study the blueberry aroma. The use of PTR-ToF-MS as an MS-e-noses resulted particularly suited to generating reliable blueberry VOCs fingerprints mainly due to a reduced compound fragmentation and precise concentration estimation. The application of PTR-MS has recently been demonstrated as a powerful phenotyping tool for fruit aroma assessment in both genetic and quality-related studies. These investigations require a detailed characterization of the aroma profile of a large fruit number; thus, fast techniques such as PTR-MS are particularly suited for this application. PTR-MS was indeed successfully applied to discriminate the aroma variability in tomato (Farneti et al., 2012, 2013), apple (Cappellin et al., 2012b; Farneti et al., 2015b, 2017), strawberry (Granitto et al., 2007), raspberry (Aprea et al., 2009), pepper (Taiti et al., 2015). Therefore, headspace VOCs fingerprint by PTR-MS provides a potential tool for discriminating blueberry fruit not only based on genetic differences but also based on origin and maturity stages.

On the other side, a weak aspect of this technology is still represented by compound identification. PTR-ToF-MS separates many blueberry isobaric compounds; however, isomers are still not distinguishable, because only the empirical formula of a compound can be determined from accurate mass data. When the formula has been identified, the step toward compound identification might not be trivial. Fragmentation, complex peak structure, and/or the presence of isomeric compounds may still make the chemical identification unpractical, especially in complex matrices. In particular, the link between PTR-ToF-MS peaks and SPME-GC-MS data of the same sample, as already pointed out by Cappellin et al. (2012a), is generally not obvious, meaning that a one-to-one relation is in general not expected, because of the presence of residual fragmentation and isobaric compounds (PTR-ToF-MS data) and of the semiquantitative analysis allowed by SPME fiber. In this study, most of the PTR-ToF-MS peaks detected in blueberry fruit were putatively identified based on PLS regression analysis. Unlike traditional multiple regression models, PLS is not limited to uncorrelated variables and one of its advantages is that it can handle noisy, collinear, and missing variables However, the *in silico* fragmentation of the compounds previously detected by SPME-GC-MS, and the fragmentation analysis of commercial standards were crucial to get rid of all incorrect attribution based only upon PLS correlations.

In our opinion the road map for flavor improvement of blueberry fruit is still at an early stage. A better understanding of the mechanisms controlling the synthesis of aroma volatiles in blueberry could provide us the ability to manipulate blueberry fruit to optimize flavor at the time of consumption. Understanding properties of enzymes involved in the production of aroma volatiles may lead to genetic and environmental manipulations to improve blueberry flavor following shipping and marketing. Nevertheless, results of this comprehensive characterization revealed the complexity of blueberry aroma profile and allowed the identification of the most affecting VOCs that can be used as putative biomarkers to rapidly evaluate the aroma variations related to ripening and/or senescence as well as to genetic background differences.

## Author contributions

BF designed the research, analyzed and interpreted data, and wrote the manuscript. IK helped with measurements, processed, and analyzed PTR-ToF-MS data. MG end MA assessed the fruit quality analysis and sampled the blueberries. EB processed and analyzed SPME-GC-MS data. AA helped with samples and measurement preparation. LC and EA revised the manuscript and checked the data FG guided the SPME-GC-MS analysis and edited the manuscript. FB guided the PTR-ToF-MS analysis and edited the manuscript. LG coordinated the work design, contributed to data interpretation, and edited the manuscript. All authors approved the manuscript.

## Funding

This work was financially supported by the AdP of the PAT (Provincia Autonoma di Trento) and by the project AppleBerry (L6/99 of the PAT).

### Conflict of interest statement

The authors declare that the research was conducted in the absence of any commercial or financial relationships that could be construed as a potential conflict of interest.

## Supplementary material

Supplementary Figure 1 **PCA analysis based on textural proprieties of blueberry fruits harvested at different ripening stages**.

Supplementary Figure 2 **Bar plot of differences in pH, titratable acidity, and total soluble solids content between blueberry fruits, of five cultivars, assessed at ripe, and overripe maturity stages**.

Supplementary Figure 3 **MS detection spectra of unknown compounds detected by SPME-GC-MS analysis**.

Supplementary Figure 4 **High resolution vector form of the loading plot reported in Figure **4B****.

Supplementary Figure 5 **High resolution vector form of the PLS correlation network reported in Figure **4****.

Supplementary Figure 6 **Loading plots of the PCA analysis reported in Figure **5****.

Supplementary Table 1 **Volatile compounds identified by SPME-GC-MS sorted based on SOTA clustering**.

Supplementary Table 2 **Volatile compounds identified by PTR-ToF-MS sorted based on SOTA clustering**.

Supplementary Table 3 **ANOVA table of VOC differences between Vaccinium species assessed by PTR-ToF-MS**.
