# Supplementary figures and images for: Exploring Blueberry Aroma Complexity by Chromatographic and Direct-Injection Spectrometric Techniques
Source: Front Plant Sci. 2017 Apr 26;8:617. doi: 10.3389/fpls.2017.00617 (PMC5405137; doi:10.3389/fpls.2017.00617)

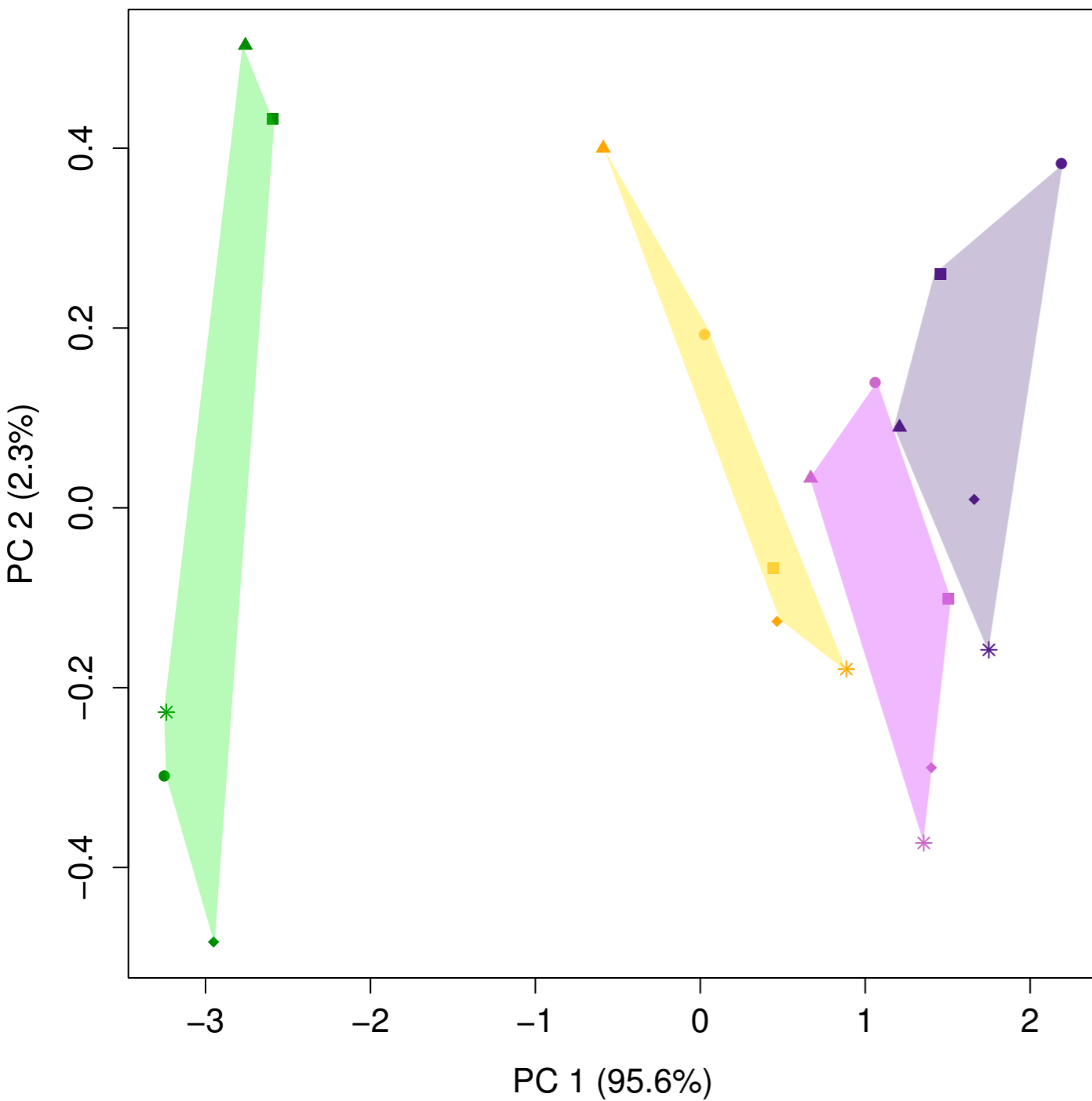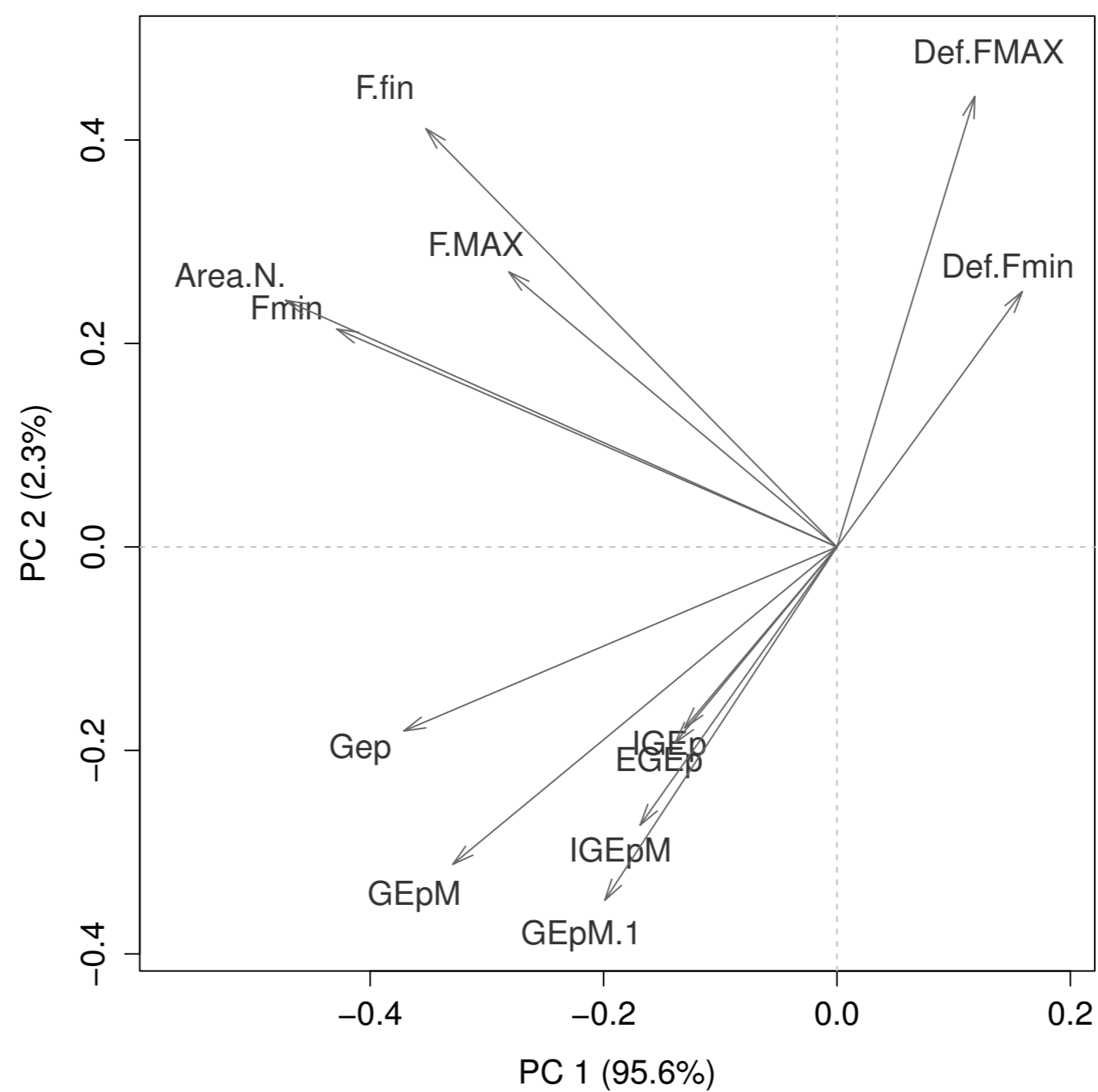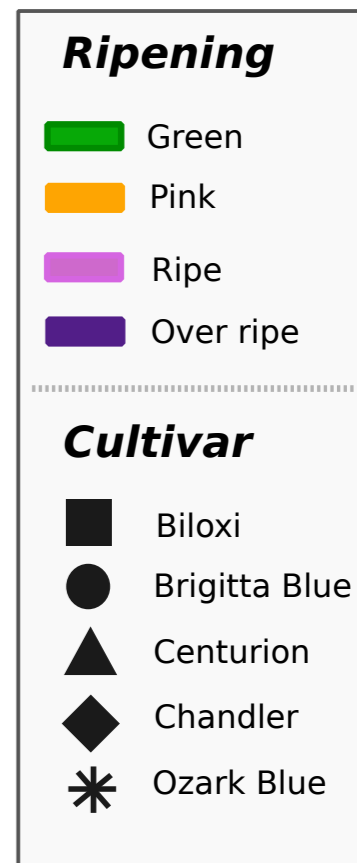

Supplement: Supplementary Figure 1 — PCA analysis based on textural proprieties of blueberry fruits harvested at different ripening stages. [file Image1.PDF]

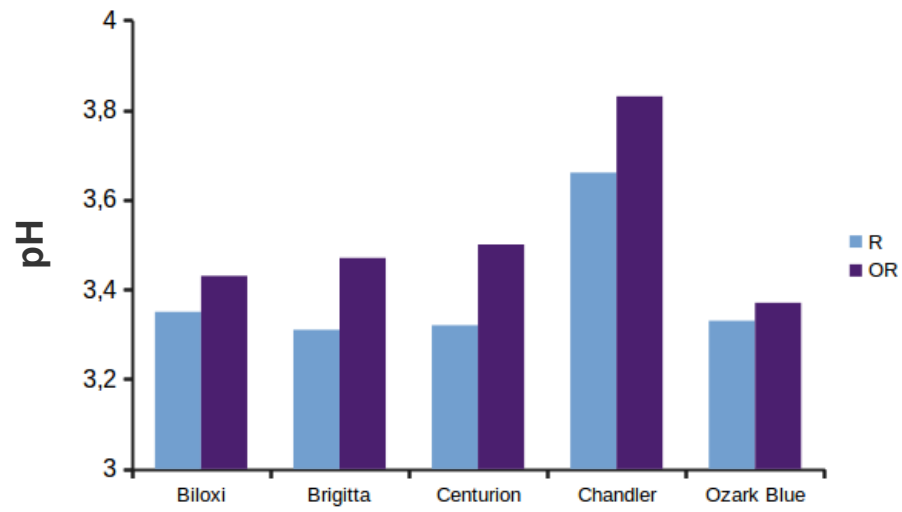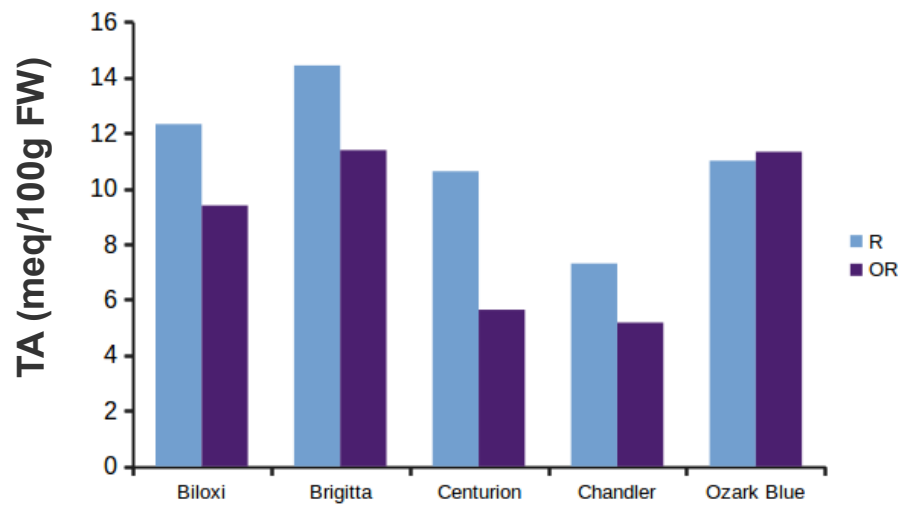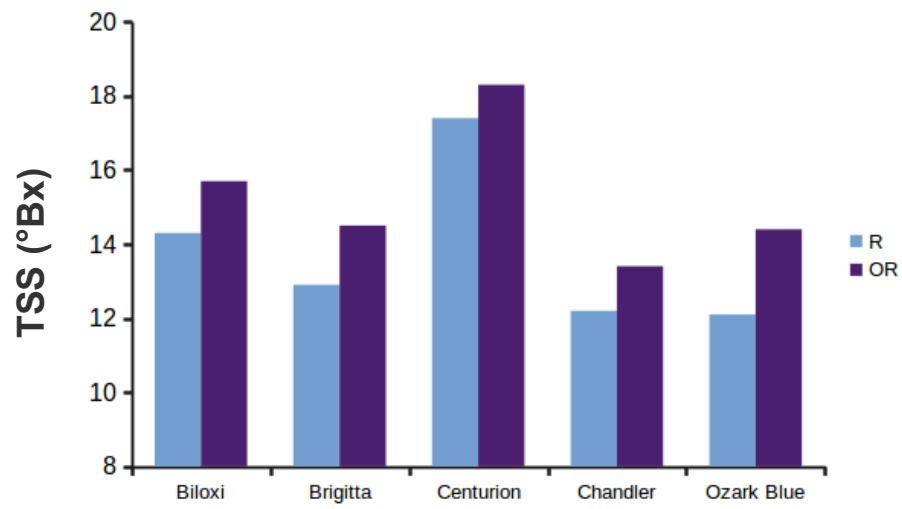

Supplement: Supplementary Figure 2 — Bar plot of differences in pH, titratable acidity, and total soluble solids content between blueberry fruits, of five cultivars, assessed at ripe, and overripe maturity stages. [file Image2.PDF]

## Unknown 1 RT 7.990

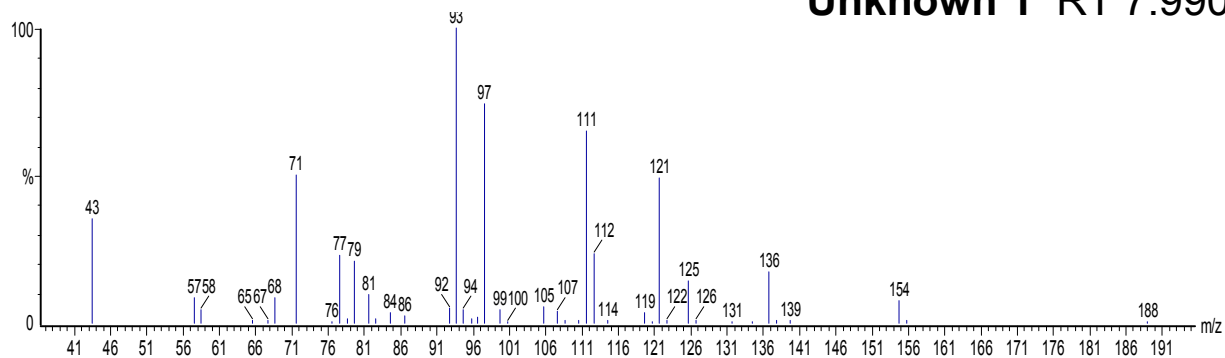

## Unknown 2 RT 10.181

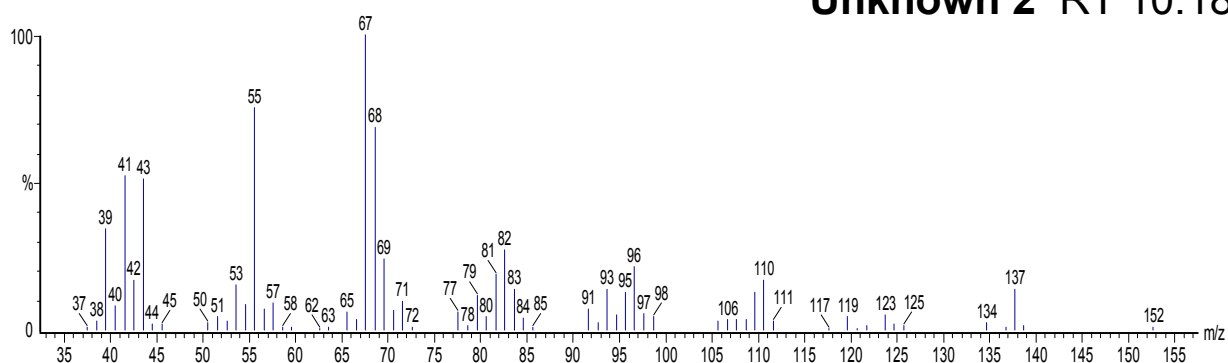

## Unknown 3 RT 12.268

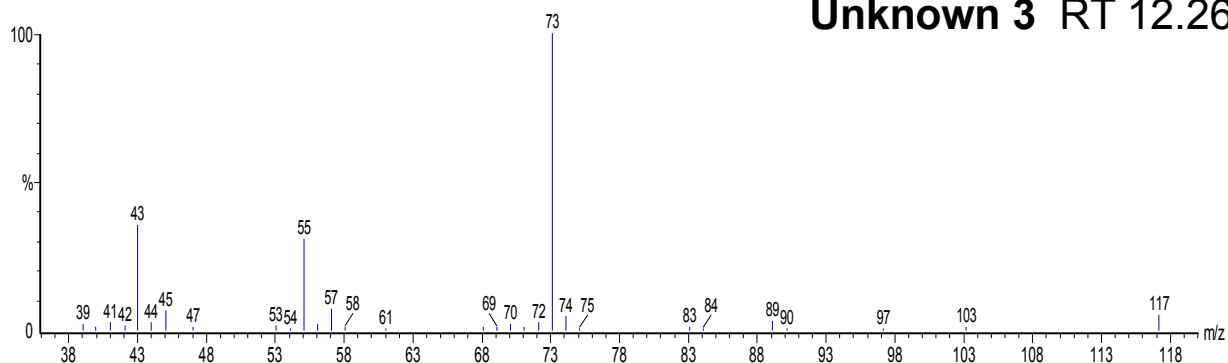

### Unknown 4 RT 12.569

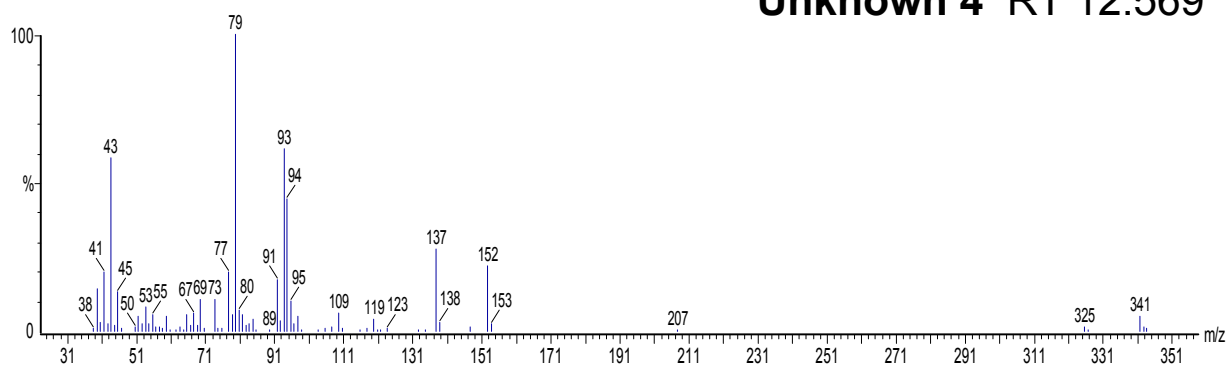

### Unknown 5 RT 19.182

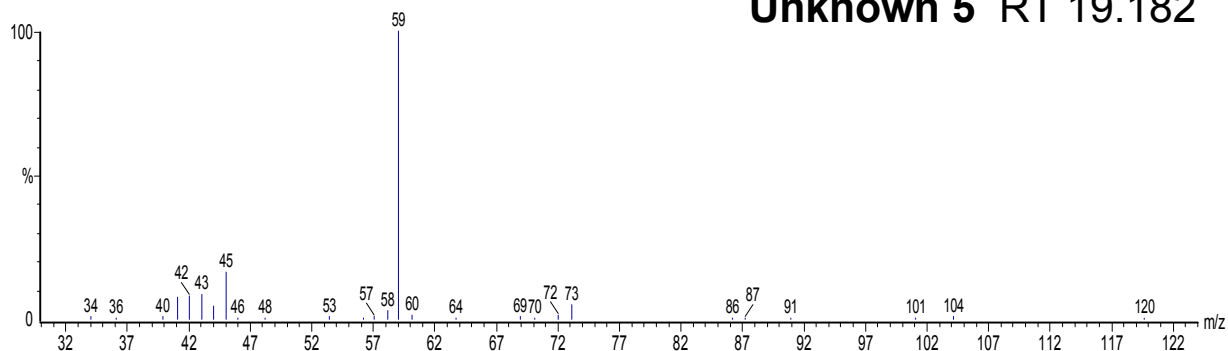

### Unknown 6 RT 31.362

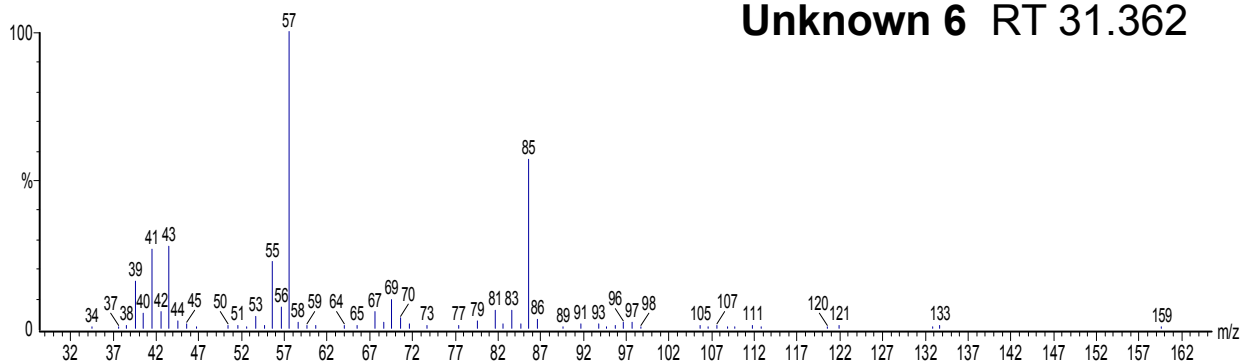

Supplement: Supplementary Figure 3 — MS detection spectra of unknown compounds detected by SPME-GC-MS analysis. [file Image3.PDF]

PC2 18%

PC1 43%

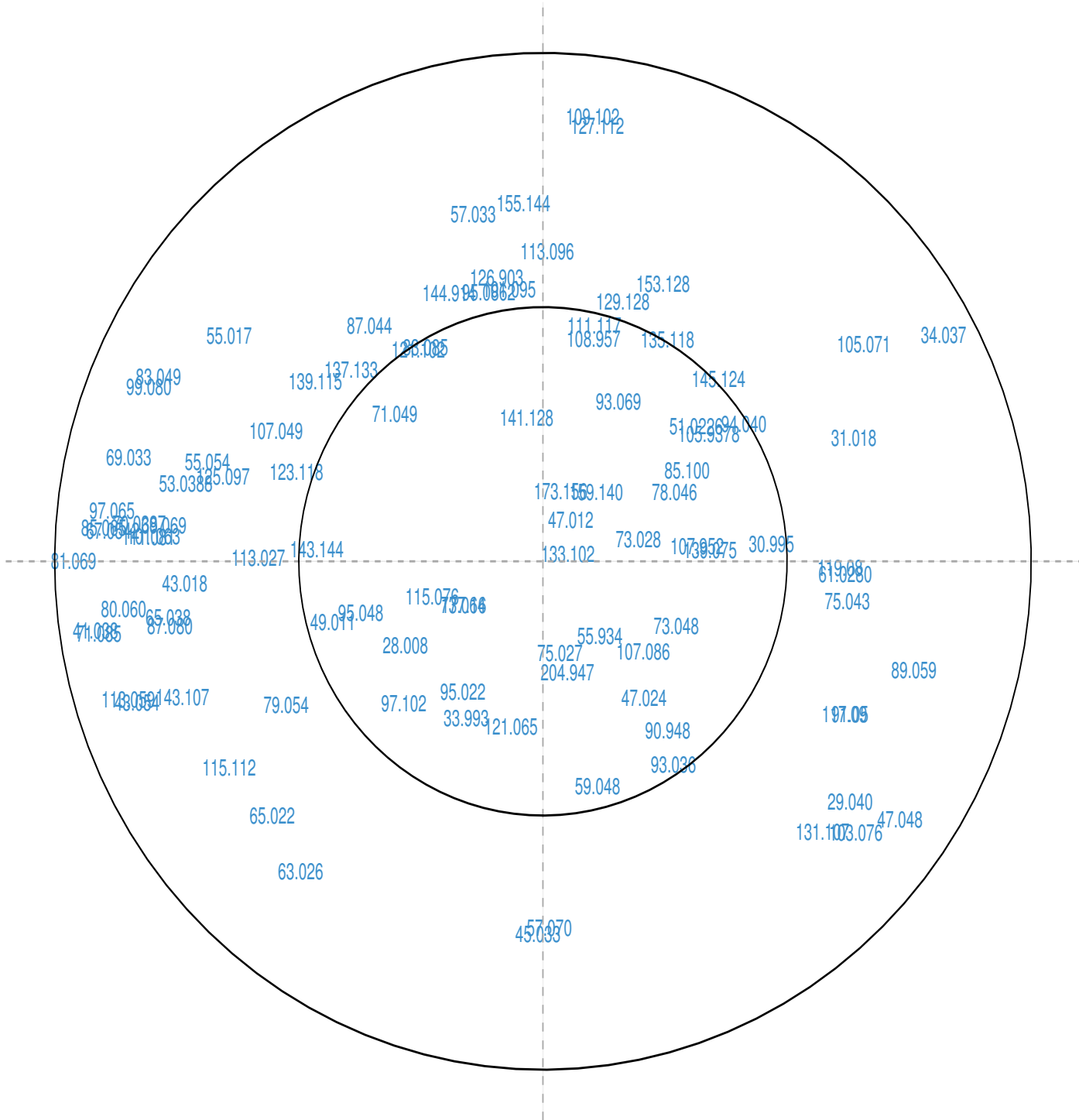

Supplement: Supplementary Figure 4 — High resolution vector form of the loading plot reported in Figure 4B. [file Image4.PDF]

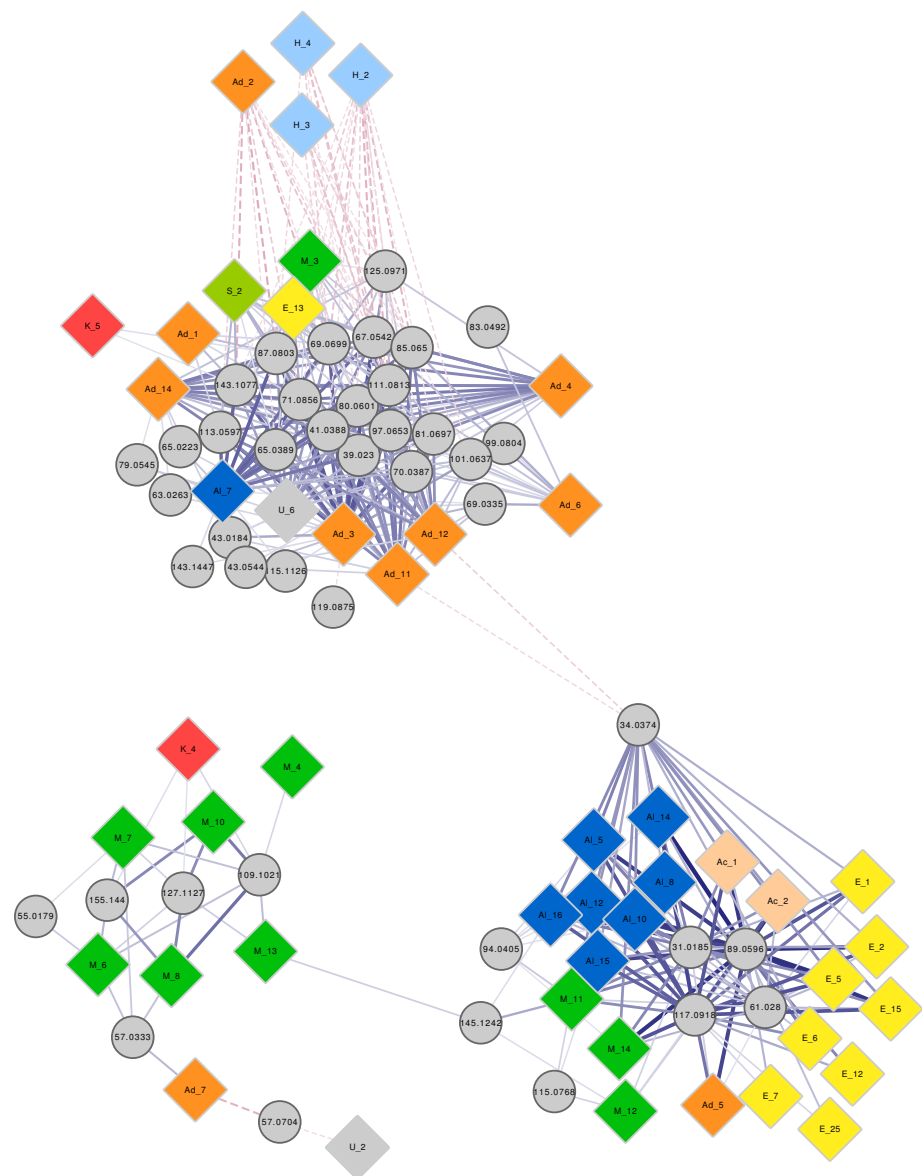

Supplement: Supplementary Figure 5 — High resolution vector form of the PLS correlation network reported in Figure 4. [file Image5.PDF]

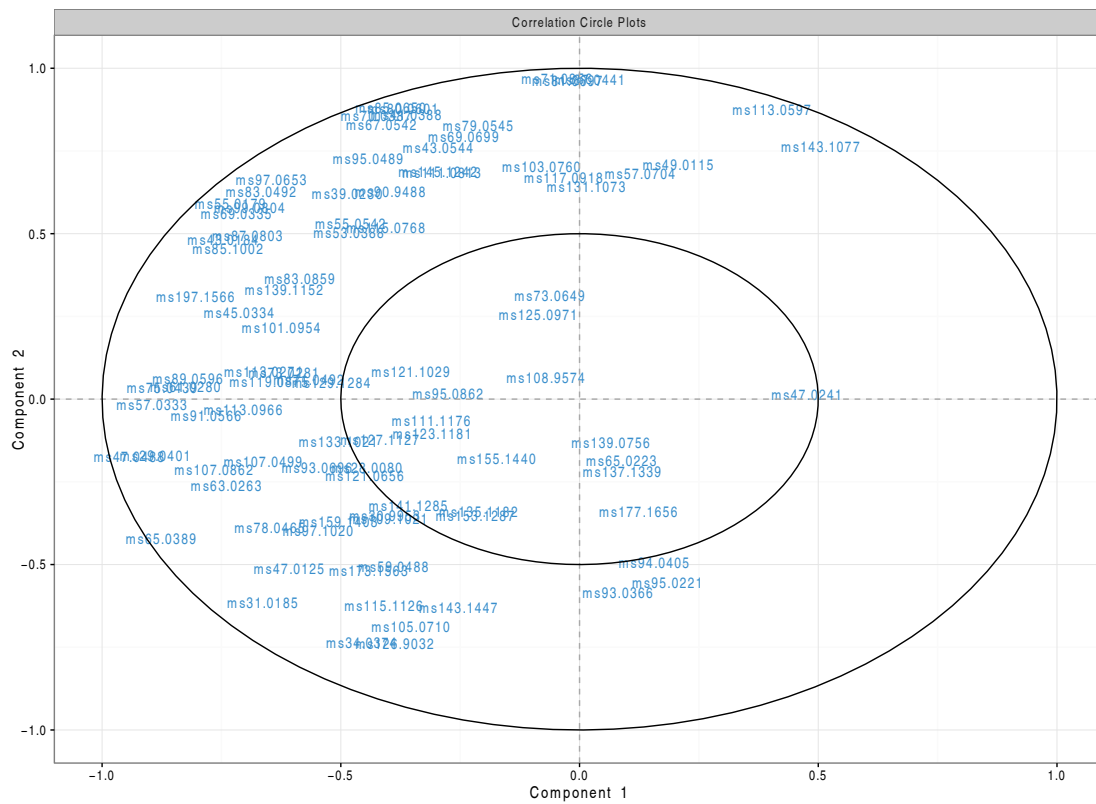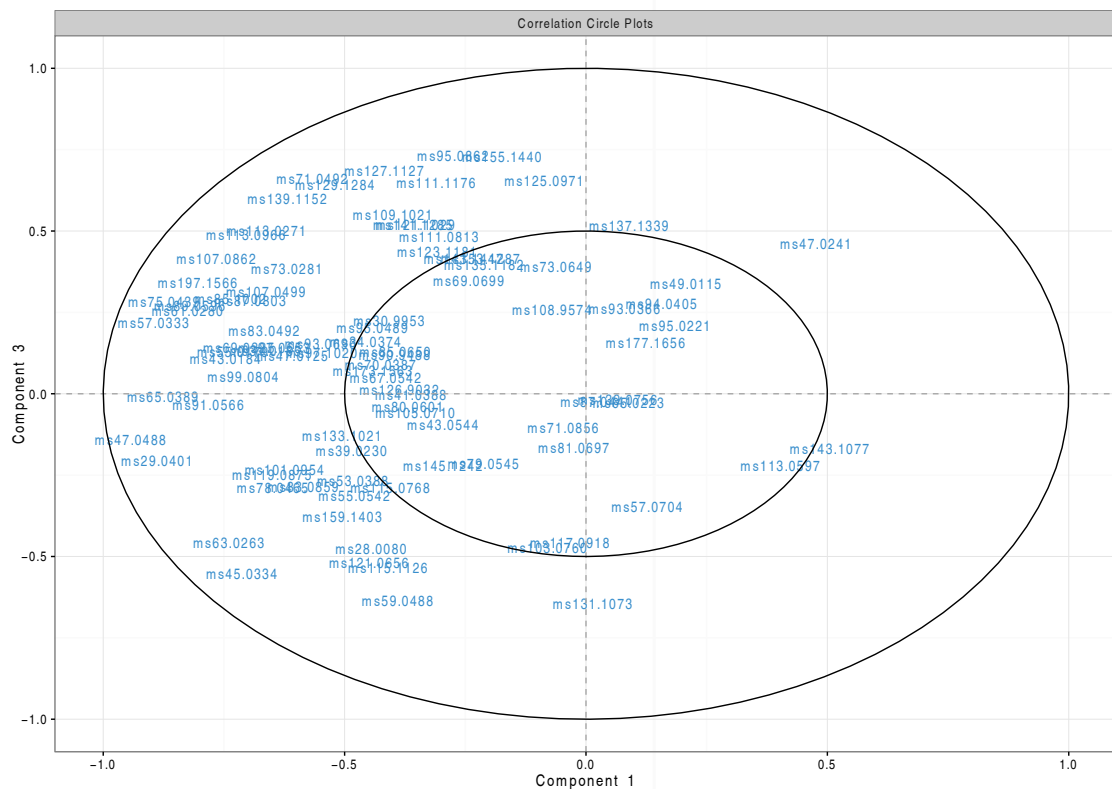

Supplement: Supplementary Figure 6 — Loading plots of the PCA analysis reported in Figure 5. [file Image6.PDF]
